# Comparative genomics of two closely related *Acropora* coral species with different spawning seasons reveals genomic regions possibly associated with gametogenesis

**DOI:** 10.1186/s12862-025-02432-5

**Published:** 2025-09-01

**Authors:** Shiho Takahashi-Kariyazono, Akira Iguchi, Yohey Terai

**Affiliations:** 1 National Institute of Advanced Industrial Science and Technology (AIST), Geological Survey of Japan, Tsukuba, Ibaraki Japan; 2 Research Center for Integrative Evolutionary Science, SOKENDAI (The Graduate University for Advanced Studies), Shonan Village, Hayama, Kanagawa Japan; 3 Research Laboratory on Environmentally-Conscious Developments and Technologies, National Institute of Advanced Industrial Science and Technology, Ibaraki, 305-8567 Japan

**Keywords:** MTORC1, Oogenesis, Cnidaria

## Abstract

Marine invertebrates release their gametes at an optimal time to produce the next generation. In reef-building scleractinian corals, synchronous spawning is essential for reproductive success. Molecular mechanisms of scleractinian gametogenesis have been studied; however, the mechanism by which coral gametes mature at specific times has yet to be discovered. The present study focused on two *Acropora* species with different spawning seasons. In Okinawa, Japan, *Acropora digitifera* spawns from May to June, whereas *Acropora* sp. 1 spawns in August. Comparative genomic analyses revealed that 60 genes are located in the diverged genomic regions between the two species, suggesting a possible association with timing of gametogenesis. Among candidate genes, we identified an *Acropora* sp. 1-specific amino acid change in gene *WDR59*, one of the components of a mTORC1 activator, GATOR2. Since regulation of gametogenesis by mTORC1 is widely conserved among eukaryotes, the difference in timing of gamete maturation observed in the two *Acropora* species may be caused by a substitution in WDR59 that slightly affects timing of mTORC1 activation via GATOR2. In addition, this substitution may lead to reproductive isolation between the two species, due to different spawning periods. Thus, we propose that *A. digitifera* and *Acropora* sp. 1 species pair is an effective model for studying coral speciation and understanding the molecular mechanisms that control coral spawning timing.

**Supplementary Information:**

The online version contains supplementary material available at 10.1186/s12862-025-02432-5.

## Background

Reef-building (scleractinian) corals are commonly hermaphroditic and reproduce through both sexual and asexual reproduction. There are two types of sexual reproduction: spawning corals that release both eggs and sperm into the water column (referred to as spawning) for external fertilization, and brooding corals that release only sperm, which are then taken up by colonies for internal fertilization. Most spawning corals reproduce sexually only once annually and do not generally self-fertilize successfully [1]. It has been demonstrated that sperm concentration is crucial for coral fertilization success [2], and gamete density in the surrounding environment during fertilization is likely affected by the synchrony of spawning [3], water flow patterns [3], and the density of conspecific coral population [4]. Then synchronized gamete maturation and gamete release are crucial for enhancing fertilization success.　For example, over 100 coral species spawn in the Great Barrier Reef between the full and last quarter moon in late spring [5, 6]. At locations such as Sesoko Island in Okinawa, Japan, where the coral species studied in this research inhabit, over 50 coral species participate in synchronized spawning, and some phylogenetic patterns regarding the timing of peak spawning nights have been reported [7].

It is known that coral gametogenesis is influenced by coral colony size (age) and external environmental factors [8]. Generally, synchronized spawning in corals is divided into three stages: (1) spawning month, (2) spawning day, and (3) spawning time. It is considered that seasonal water temperature variations are involved in determining the spawning month [9, 10], lunar cycles and water temperature influence the spawning day [5, 11], and sunset stimuli regulate the spawning time [5, 12]. In several coral species, spawning has become asynchronous due to the effects of recent climate change [13]. Therefore, understanding the mechanisms of synchronous gamete maturation will help us estimate the impact of climate change on coral reproduction and restoration using coral seedlings produced from gametes [14].

Gametogenesis in corals has been studied in the field [15] and by molecular biological approaches [16, 17]. Histological observations of gametogenesis have revealed that oocytes are observed 11 months before the spawning month, clusters of spermatogonia appear approximately 6 months prior, meiosis begins 2 − 1 months before spawning, and maturation occurs several weeks before spawning [16, 18]. Additionally, transcriptome analysis of reproductive tissues has revealed that coral gametogenesis shares conserved molecular characteristics of gametogenesis found across metazoans [17]. The progression of oogenesis stages correlates with the changes in sea surface temperature and photoperiod [18]. Coral gametogenesis is dependent on photosynthetic carbon derived from endosymbiotic　photosynthetic microalgae　Symbiodiniaceae [19], while environmental conditions such as water temperature and photoperiod influence Symbiodiniaceae photosynthetic activity. Based on these findings, environmental conditions, including photoperiod and water temperature, may serve as determinants of coral gametogenesis through their effects on the nutritional status of corals derived from Symbiodiniaceae [18]. However, the molecular mechanisms by which environmental factors affect the progression of gametogenesis remain unclear.

In the Indo-Pacific region, including Okinawa, Japan, the genus *Acropora* comprises the largest number of coral species [20]. In Okinawa, most *Acropora* species spawn around the full moon in May or June, with a few species spawning several months later [21]. One species that spawns later is *Acropora* sp. 1. This species was initially classified as *Acropora digitifera* [22]; however, the two are now recognized as separate species, due to differences in morphology and spawning time [23–25]. *Acropora* sp. 1 has a flatter colony shape and shorter branches than *A. digitifera* [23, 24]. *Acropora* sp. 1 tends to inhabit reef edges with faster (offshore) currents than *A. digitifera*. In addition, in Okinawa, *A. digitifera* spawns from May to June, whereas *Acropora* sp. 1 spawns in August [23]. Histological observations of *A. digitifera* and *Acropora* sp. 1 indicate that *Acropora* sp. 1 shows a delay not only in spawning month but also in the timing of egg maturation onset compared to *A. digitifera* [23]. Gametes of both species can cross-fertilize as indicated by artificial fertilization experiments [24]. Under natural conditions, however, the two species do not interbreed because of the different spawning months [24].

Advances in analysis of genomic data with next-generation sequencers have revealed the genetic basis of specific traits [26]. In particular, comparative genomic analyses between genetically close species have identified genomic regions associated with their phenotypic differences [27, 28]. So far, genomes of various corals have been sequenced [29–32], and population genomic approaches have identified loci associated with heat tolerance [33]. Comparative genomic analysis has yet to be conducted to identify genomic regions associated with differences in coral spawning timing due to the lack of closely related species pairs to compare.

In this study, we performed a comparative genomic analysis between *A. digitifera* and *Acropora* sp. 1 to identify genomic regions likely involved in trait differences between them. We expected that *A. digitifera* and *Acropora* sp. 1 were genetically closely related based on analysis of short sequences [25] and their fertilization ability [24]. Therefore, we determined the genome sequences of *Acropora* sp. 1. This comparative genomic analysis identified genomic regions likely associated with differences in their timing of egg maturation and spawning month. Since differences in spawning time can lead to reproductive isolation, these species will be a useful model to study coral speciation and to understand molecular mechanisms that regulate spawning time in corals.

## Methods

### Specimen collection and species identification

Coral samples were collected from two reefs at Okinawa, Japan, between 2018 and 2020 (Table S1) with permission of the Aquaculture Agency of Okinawa Prefecture (permit numbers 30 − 29, 31–43, and 31–68). Sixteen colonies of *Acropora* sp. 1 with visible gametes, were collected in the field and subsequently maintained in an aquarium at the Sesoko Station, Tropical Biosphere Research Center, University of the Ryukyus. In 2018, gametes of one *Acropora* sp. 1 colony were collected during spawning, and sperm were preserved at −80 °C until genome extraction. After we placed the coral colonies in the aquarium, we preserved branch fragments in RNAlater (Waltham, MA, USA) for genome extraction in 2019 and 2020.

### DNA extraction and sequencing

We extracted genomic DNAs from 15 branch fragments originating from 15 *Acropora* sp. 1 colonies using a DNeasy Plant Mini Kit (QIAGEN, Hilden, Germany). We used DNeasy Blood & Tissue Kits (QIAGEN, Hilden, Germany) for DNA extraction from sperm originating from one *Acropora* sp. 1 colony. Following the manufacturer’s instructions, we constructed DNA libraries from 16 samples using an NEBNext Ultra II DNA Library Prep Kit (Illumina). The 15 libraries from branch tissues were sequenced on an Illumina HiSeqX Ten, and one library from sperm was sequenced on an Illumina HiSeq 2500.

### Mapping and variant calling

For *A. digitifera*, we selected genome re-sequence data from previously published resequencing data of adult *A. digitifera* colonies collected from the Ryukyu Archipelago. We downloaded genome sequence data from 11 colonies of *A. digitifera* and 15 *Acropora* species (*A. tenuis*,* A.yongei*,* A. intermedia*,* A. gemmifera*,* A. awi*,* A. florida*,* A. millepora*,* A. selago*,* A. hyacinthus*,* A. cytherea*,* A. muricata*,* A. echinata*,* A. acuminata*,* A. nasuta*,* and A. microphthalma*) from the DNA Data Bank of Japan (DDBJ) (accession IDs are shown in Tables S1and S2). We trimmed raw sequences and removed low-quality reads before mapping with fastp [34]. Trimmed reads were mapped to the *A. digitifera* genome assembly ver. 2.0 [30] using bowtie2 ver. 2.3.3.1 [35] without repetitive sequences masking. Among 16 *Acropora* sp. 1 colonies, we used 14 colonies with mapping bam coverage ≥ 10 for variant calling. Variants were called using Genome Analysis Toolkit (GATK) version 4.0 and filtered according to a GATK-suggested hard-filtering with a minor modification.

### PCA and molecular phylogenetic tree construction

For phylogenetic analysis using IQ-TREE 3 [36], we used 798,399 variable sites from 17 *Acropora* species: 14 individuals for *Acropora* sp. 1, 11 individuals for *A. digitifera*, and one individual each for the remaining 15 *Acropora* species. For the IQ-TREE 3 [36] analysis, we used the GTR + ASC model. We performed bootstrap analysis with 1,000 replicates to assess branch support and conducted site concordance analysis with 100 replicates to evaluate the concordance of individual sites with the inferred phylogeny. We performed principal components analysis (PCA) on the genome-wide pruned 72,051 SNVs using PLINK v1.90 (http://pngu.mgh.harvard.edu/purcell/plink/) using a vcf file including 14 individuals for *Acropora* sp. 1, and 11 individuals for *A. digitifera.*

### Genome scan of highly diverged regions (HDRs)

We calculated F_ST_ [37] between *A. digitifera* and *Acropora* sp. 1 populations for 10-kb windows with 1 kb increments along each scaffold (> 10 kb) using a sliding window approach with PopGenome [38]. First, we extracted 10 kb windows that included the top 0.1% of F_ST_ values. Among these top windows, we selected windows with single-nucleotide variants (SNVs) for which the allele is fixed in one population (frequency = 1.0) and for which there were no homozygotes for that allele in the other population (heterozygotes were allowed). We considered these SNVs to be diverged SNVs. We merged adjacent and overlapping windows among these selected windows into continuous genomic regions and considered these merged regions as highly diverged (Table S3).

### Identification of genes in HDRs

We considered genes with diverged SNVs in HDRs as candidate genes related to phenotypic differences between the *A. digitifer* and *Acropora* sp. 1. To identify functional annotations of these genes, we searched putative orthologs of 60 candidate genes using phylogenetic based Phylogenetic based OrthoFinder. Briefly, we performed ortholog inference using Phylogenetic based OrthoFinder with transcripts from *A. digitifera* and the following four model organisms: yeast (*Saccharomyces cerevisiae*), fruit fly (*Drosophila melanogaster*), zebrafish (*Danio rerio*) and human (*Homo sapiens*). We used PANTHER (https://pantherdb.org) to search for the annotation orthologous genes corresponding to each *A. digitifera* candidate gene in model organisms and compiled the ortholog information (PANTHER Family/Subfamily) from the model organisms. For genes for which orthologs were not identified in any of the four model organisms, we searched homologous genes in the NCBI nucleotide database by Blast search(https://blast.ncbi.nlm.nih.gov/Blast.cgi) [39]. We regarded the top hit with an *e*-value ≥ 1e^–30^ and identity ≥ 90% for NCBI as a homologous gene (Table S4). We determined whether diverged SNVs cause amino acid changes using CLC Genomics Workbench 11.0 (QIAGEN, Aarhus, Denmark) (Table S5).

### Identification of a deletion in WDR59 among *Acropora* sp. 1

The presence of one deletion in the *WDR59* gene in *Acropora* sp. 1, discovered by visual confirmation of the mapping results, was revealed by amplifying the genomic region containing the deletion using PCR and sequencing it. We used the following genomic DNAs as templates for PCR: Genomic DNAs extracted from 7 *A. digitifera* colonies (sample ID: AdigS1601–4, AdigS1606–07 and AdigS1610) and 14 *Acropora* sp. 1 colonies (sample ID: Asp1B1901-07, Asp1c, Asp1S2001-03, Asp1S2005, and Asp1S2007-08).

### WDR59 sequences among *Acropora* species

To confirm the similarity of *WDR59* between *A. digitifera* and *Acropora* sp. 1, we extracted a consensus sequence for CDS of WDR59 from short read mapping data of an *Acropora* sp.1 sample (ID: Asp1S2003) using CLC Genomics Workbench 11.0 (QIAGEN, Aarhus, Denmark). The consensus sequence is shown in the supplemental material. The evolutionary divergence between the *WDR59* sequence of *A. digitifera* (adig_s0048.g28.t1) and *Acropora* sp. 1 (extracted consensus sequence) is estimated by MEGA 7 [40]. 

To confirm the orthologous relationship of *Acropora* sp. 1 and *A. digitifera WDR59*, we constructed a phylogenetic tree of *WDR59* and *WDR24* using 14 *Acropora* species (*A. tenuis*,* A. intermedia*,* A. gemmifera*,* A. awi*,* A. florida*,* A. selago*,* A. hyacinthus*,* A. muricata*,* A. echinata*,* A. acuminata*,* A. nasuta*,* A. microphthalma*,* A. digitifera*, and *Acropora* sp. 1). Putative orthologous genes of *WDR59* and *WDR24* were searched in the reference transcriptome of each of the 15 *Acropora* species (Table S6) using Phylogenetic based OrthoFinder version 2.5.4 [41]. To avoid reducing the number of sites for phylogenetic analysis, *WDR59* sequence of *A. cytherea* (610 aa) and *A. yongei* (642 aa) were removed from the tree construction because these sequences were too short compared with *WDR59* of *A. digitifera* (964 aa). WDR24 sequence of *A. cytherea* (531 aa), *A. Florida* (675 aa), *A. yongei* (604 aa and 630 aa), and *A. tenuis* (606 aa) were removed from the tree construction because these sequences were too short compared with WDR59 of *A. digitifera* (824 aa). In total, 31 sequences from 14 *Acropora* species were used for phylogenetic construction. Gene ID used for the phylogenetic analysis were shown in Table S7 and S8.

### Gene expression levels of candidate genes

To check expression of candidate genes during spawning, we analyzed two RNA-seq datasets from published studies: *A. digitifera* [42] and *A. tenuis* [43]. The transcriptome data of *A. digitifera* (SRR3316332, SRR3316333, SRR3316336, and SRR3316337) and *A. tenuis* (DRR288026-DRR288034) were used for calculation of gene expression value (Transcripts per million: TPM) by CLC Genomics Workbench 11.0 (QIAGEN, Aarhus, Denmark). The first dataset was the transcriptome of *A. digitifera* during spawning published in 2017 by Rosenberg et al. [42]. We analyzed four pooled RNA-seq data (three colonies at each time point) to confirm gene expression values at specific reproductive stages for *WDR59* and Related Pathway Components: pre-spawning, setting (approximately 10–20 min before spawning), egg-sperm bundle release (spawning), and post-spawning. Because these data are from a pooled-RNA library and there is no replicates for each stage, gene expression value (Transcripts per million: TPM) of candidate genes were listed without comparing gene expression levels at each stage. The second dataset was the transcriptome of *A. tenuis* during the spawning season published in 2022 by Takekata et al. [43]. We analyzed nine transcriptomes from the two *A. tenuis* colonies (C2 and C3) at three time points: May (pre-spawning), June (pre-spawning), and July (post-spawning) using the genome assembly of *A. tenuis* as a reference genome. We did not analyzed transcriptome of one colony (C1), because according to Takekata et al. 2022 [43], one colony (C1) was immature during their observation and did not spawn. Since there were two colonies excluding the immature colony and the number of replicates for each month was two, the gene expression values (TPM) of the candidate genes were described without comparing gene expression levels at each stage. Putative orthologs in *A. tenuis* of the 60 candidate genes identified in *A. digitifera* were identified from Phylogenetic based OrthoFinder results using transcriptomes of 13 *Acropora* species (*A. tenuis*,* A. intermedia*,* A. gemmifera*,* A. awi*,* A. florida*,* A. selago*,* A. hyacinthus*,* A. muricata*,* A. echinata*,* A. acuminata*,* A. nasuta*,* A. microphthalma*, and *A. digitifera*) as described in the aforementioned “WDR59 sequences among *Acropora* species” section. The percentile rank of WDR59 TPM relative to all expressed genes was calculated.

## Results

### The spawning month of *Acropora* sp. 1

**Fig. 1:**
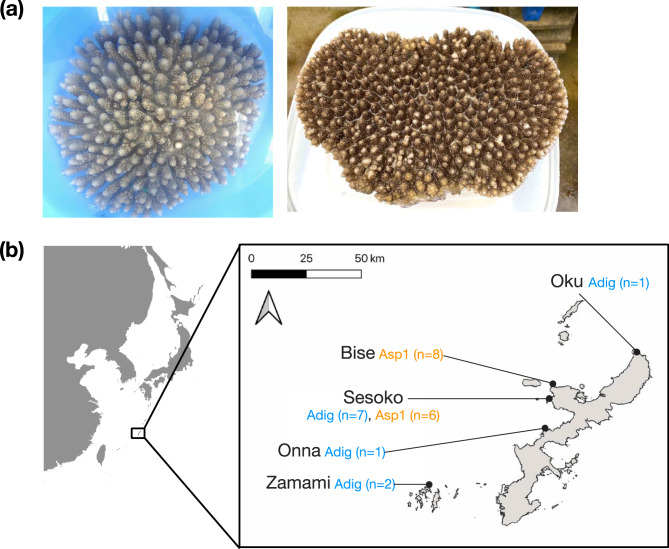
**a** Adult colonies of *Acropora digitifera* (left) and *Acropora* sp. 1 (right). **b** Sampling locations are shown as dots on the map of Okinawa Island

We collected 16 *Acropora* sp. 1 colonies during 2018–2020 at Sesoko and Bise, Okinawa, Japan (Fig. 1), and observed mature oocytes or spawning in August (Table S1). This later-spawning month of *Acropora* sp. 1 is consistent with previous observations [23–25].

### Phylogenetic tree and principal component analysis

**Fig. 2:**
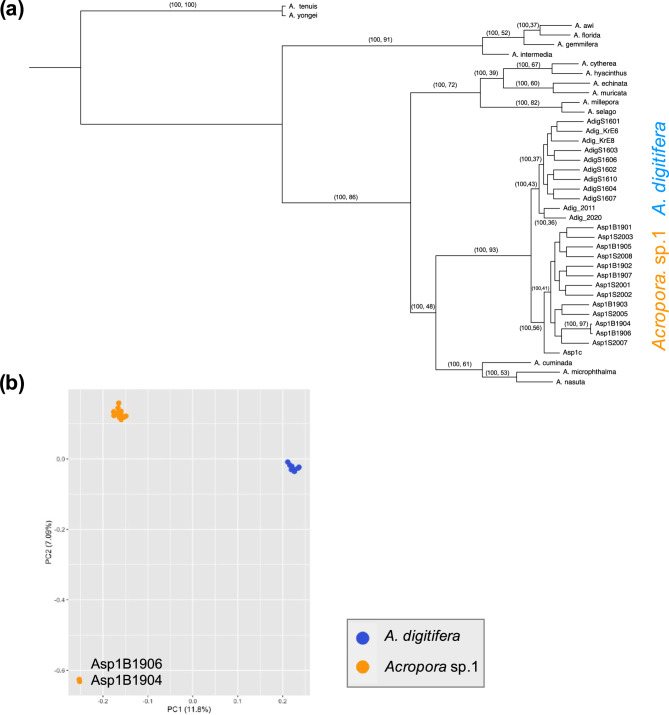
Phylogenetic relationship of *Acropora* sp. 1 (**a**) Phylogenetic relationships of 17 *Acropora* corals were analyzed based on 798,399 SNVs using the maximum likelihood method with GTR + ASC model. Bootstrap support and site concordance shown next to each node for each clade. **b** PC1 and PC2 were derived from PCA based on 72,051 SNVs for all individuals of *A. digitifera* and *Acropora* sp. 1

First, we investigated the genetic relationship between *A. digitifera* and *Acropora* sp. 1　using 798,399 SNVs extracted from 11 *A. digitifera* colonies, 14 *Acropora* sp. 1 colonies, and one colony each from 13 *Acropora* species (*A. tenuis*,* A. intermedia*,* A. gemmifera*,* A. awi*,* A. florida*,* A. selago*,* A. hyacinthus*,* A. muricata*,* A. echinata*,* A. acuminata*,* A. nasuta*,* A. microphthalma*, and *A. millepora*). *A. digitifera* and *Acropora* sp. 1 colonies formed a monophyletic clade supported by bootstrap support of 100 and a site concordance factor of 93 (Fig. 2a). In this clade, *A. digitifera* and *Acropora* sp. 1 colonies each formed monophyletic clades with bootstrap support of 100 but a site concordance factor of only 43, while the *Acropora* sp. 1 lineage had a site concordance factor of 56 (Fig. 2a). We performed principal component analysis (PCA) using 11 *A. digitifera* colonies and 14 *Acropora* sp. 1. *A. digitifera* colonies were separated from *Acropora* sp. 1 along the PC1 axis (Fig. 2b). Among *Acropora* sp. 1 colonies, two (Colony IDs: Asp1B1906 and Asp1B1904) were separated from other *Acropora* sp. 1 colonies along the PC2 axis (Fig. 2b). In addition, these two colonies (Colony IDs: Asp1B1906 and Asp1B1904) formed a single clade with high bootstrap support in the phylogenetic tree (Fig. 2a). These two colonies were sampled from the same sites as other colonies sampled in the same year, indicating no geographic isolation.

### Highly diverged regions between *A. digitifera* and* Acropora* sp. 1

**Fig. 3:**
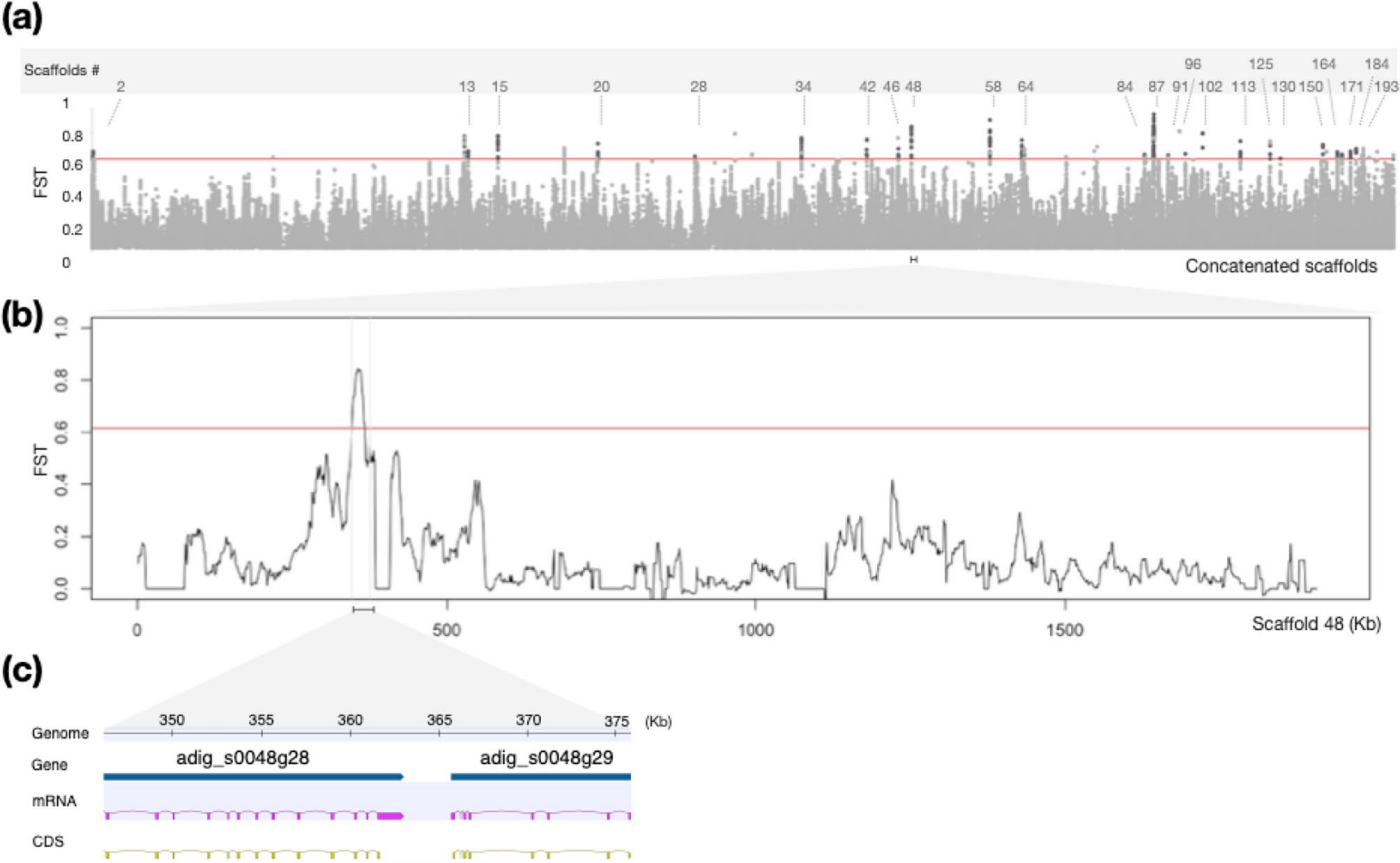
The genome-wide pattern of genetic differences between the two species. **a** Genome-wide FST values were calculated in overlapping windows of 10 kb. The red line indicates the top 0.1% of values. **b** FST was estimated across a region of scaffold 48 (adig_s0048). The red line indicates the top 0.1% of values. **c** A close-up view of predicted gene structures on an HDR in scaffold 48 (adig_s0048). The flanking gene structure of WDR59 (Gene ID: adig_s0048.g28) and guanine nucleotide-binding protein G(o) subunit alpha (Gene ID: adig_s0048.g29) are indicated

The degree of genetic differentiation among subpopulations is measured by F_ST_ [44, 45]. Since phylogenetic analysis indicated that *A. digitifera* and *Acropora* sp. 1 are closely related, we calculated F_ST_ [46] between two species using 1,459,328 SNVs. The F_ST_ [46] value across the genomes of these two species was 0.10225. This is comparable to the genetic divergence of species pairs used in comparative genome analysis in previous studies [47–49]. Despite low genetic divergence throughout their genomes, genomic regions responsible for differences in traits between *A. digitifera* and *Acropora* sp. 1 are expected to differ in the two species. To extract diverged regions, we performed a sliding window analysis of 10 kb in 1 kb increments between *A. digitifera* and *Acropora* sp. 1. Genomic regions with the top 0.1% F_ST_ [37] values (F_ST_ >0.6157) in each 10 kb window were then selected (Fig. 3a). We further selected windows containing diverged SNVs (Materials and Methods) from the top 0.1% F_ST_ [37] windows. When these windows overlapped, they were combined. As a result, 34 genomic regions, called highly diverged regions (HDRs) (Table S3), were extracted from the whole genome. We present an example of HDR in Fig. 3b. The red line indicates the top 0.1% of values, and one HDR is shown flanked by gray lines on a scaffold. This HDR region contains two genes (Fig. 3c).

### Genes in highly diverged regions

The HDRs contain 60 genes. We searched putative orthologs of 60 candidate genes in four species: *Saccharomyces cerevisiae*,* Drosophila melanogaster*,* Danio rerio*, and *Homo sapiens* (Table 1 and Figure S1). As a result, among the 60 genes, 6 genes had putative orthologs in all four species (*Saccharomyces cerevisiae*,* Drosophila melanogaster*,* Danio rerio*, and *Homo sapiens*), 10 genes had putative orthologs in *Drosophila melanogaster*,* Danio rerio*, and *Homo sapiens*, 15 genes did not meet the aforementioned two criteria but had putative orthologs in at least one of the three model organisms (*Drosophila melanogaster*,* Danio rerio*,* and Homo sapiens*), and 29 genes had no orthologs in any of the four model organisms. The *WDR59* had putative orthologs in all four species: *Saccharomyces cerevisiae*,* Drosophila melanogaster*,* Danio rerio*, and *Homo sapiens*. For genes for which orthologs were not identified in any of the four model organisms, we searched homologous genes in the NCBI nucleotide database by Blast search [39] (Table S4).

**Table 1:** Genes in highly diverged regions

Ortholog status	*A. digitifera* gene IDs	PANTHER Family/Subfamily of putative orthologs
Putative orthologs exist in yeast fruit fly zebrafish human	adig_s0002.g67.t1	NIPPED-B-LIKE PROTEIN (PTHR21704:SF18)
	adig_s0002.g69.t1	WD REPEAT-CONTAINING PROTEIN 18 (PTHR18763:SF0)
	adig_s0048.g28.t1	GATOR COMPLEX PROTEIN WDR59 (PTHR46170:SF1)
	adig_s0048.g29.t1	GTP-BINDING PROTEIN ALPHA SUBUNIT (PTHR10218)
	adig_s0064.g89.t1	PKSB (PTHR24322)
	adig_s0181.g15.t1	PROTON-COUPLED ZINC ANTIPORTER SLC30A1 (PTHR45820:SF1), ZINC TRANSPORTER 63 C, ISOFORM F (PTHR45820:SF4)
Putative orthologs exist in fruit fly zebrafish human	adig_s0028.g24.t1	INSULIN-LIKE GROWTH FACTOR 2 MRNA-BINDING PROTEIN 2 (PTHR10288:SF93),INSULIN-LIKE GROWTH FACTOR 2 MRNA-BINDING PROTEIN 3 (PTHR10288:SF158), IGF-II MRNA-BINDING PROTEIN, ISOFORM L (PTHR10288:SF338)
	adig_s0064.g91.t1	SURVIVAL OF MOTOR NEURON-RELATED-SPLICING FACTOR 30 (PTHR13681:SF26)
	adig_s0065.g70.t1	NUCLEOPORIN NUP37 (PTHR22806:SF0)
	adig_s0065.g71.t1, adig_s0065.g72.t1	ACHAETE-SCUTE TRANSCRIPTION FACTOR-RELATED (PTHR13935)
	adig_s0091.g24.t1	HOMEOBOX PROTEIN SIX (PTHR10390)
	adig_s0042.g173.t1,adig_s0042.g174.t1	TYROSINE-PROTEIN KINASE RECEPTOR (PTHR24416)
	adig_s0184.g18.t1	ARRESTIN DOMAIN CONTAINING PROTEIN (PTHR11188)
	adig_s0184.g20.t1	RHO GTPASE-ACTIVATING PROTEIN 39 (PTHR45876:SF1),
Putative orthologs exist in at least one of the following species fruit fly zebrafish human	adig_s0184.g19.t1	MICROTUBULE-ASSOCIATED PROTEINS 1 A/1B LIGHT CHAIN 3-RELATED (PTHR10969)
	adig_s0002.g57.t1	TGF-BETA FAMILY (PTHR11848)
	adig_s0064.g92.t1	GNAT FAMILY N-ACETYLTRANSFERASE (PTHR13947)
	adig_s0020.g143.t1	SIMILAR TO ENSANGP00000010363 (PTHR22930:SF279),GH03217P-RELATED (PTHR22930:SF85)
	adig_s0164.g11.t1, adig_s0164.g12.t1, adig_s0164.g13.t1, adig_s0164.g14.t1	ALPHA-(1,3)-FUCOSYLTRANSFERASE C-RELATED (PTHR48438), ALPHA- 1,3 -FUCOSYLTRANSFERASE (PTHR11929)
	adig_s0164.g40.t1	SHORT STOP, ISOFORM H (PTHR23169:SF23),MICROTUBULE-ACTIN CROSS-LINKING FACTOR 1, ISOFORMS 1_2_3_4_5 (PTHR23169:SF25)
	adig_s0015.g101.t1	RNA-BINDING PROTEIN 3 (PTHR48034:SF12)
	adig_s0084.g33.t1	UDP-N-ACETYLGLUCOSAMINE–PEPTIDE N-ACETYLGLUCOSAMINYLTRANSFERASE SPINDLY-RELATED (PTHR44858:SF1)
	adig_s0087.g3.t1	POGO TRANSPOSABLE ELEMENT WITH KRAB DOMAIN (PTHR19303:SF74)
	adig_s0150.g20.t1	OXIDASE/PEROXIDASE (PTHR11475)
	adig_s0150.g21.t1	LD42267P (PTHR11475:SF134)
	adig_s0193.g30.t1	GOLGI INTEGRAL MEMBRANE PROTEIN 4-RELATED (PTHR22909:SF24)

We surveyed the literature related to the ortholog information (PANTHER Family/Subfamily) and annotation of homologous genes. Nucleotide sequences of a gene (Gene ID: adig_s0171.g21) was similar (Table S4) to collagen alpha chain, which is associated with skeletogenesis in *Acropora* corals [50]. The amino acid sequence of another gene (Gene ID: adig_s0048.g28) was predicted to be a putative ortholog of WD repeat-containing protein 59 (WDR59) (Table 1).

The gene expression values (TPM) calculated from two RNA-seq datasets from published studies: *A. digitifera* [42] and *A. tenuis* [43] are shown in Tables S9 and S10. In the results of the RNA-seq dataset from *A. digitifera* [43], 40 genes have relatively high expression (the rank percentile of TPM under 50) in at least one stage among the PreSpawning stage, Setting stage, Spawning stage, and Post-Spawning stage. In the results of the RNA-seq dataset from *A. tenuis*, 46 genes have relatively high expression (the rank percentile of TPM under 50) in at least one colony in May, June, and July.

To identify genes whose function is affected by diverged SNVs, we identified amino acid changes between the two species caused by diverged SNVs. Among 60 genes in the HDRs, 39 genes harbor diverged SNVs in the gene regions. Among these 39 genes with diverged SNVs, 14 had at least one amino acid change between *A. digitifera* and *Acropora* sp. 1 (Table S5). Compared with the *A. digitifera* reference genome, *Acropora* sp. 1 had three amino acid changes in *WDR59* (Gene ID: adig_s0048.g28) (Table S5). WDR59 is a component of the GTPase-activating protein toward Rags (GATOR) complex, GATOR2 [51]. In *Drosophila*, GATOR2 controls meiotic entry and oocyte development [52]. Therefore, we focused further on this gene.

### Differences in WDR59 between *A. digitifera* and *Acropora *sp. 1

To confirm the similarity of *WDR59* between *A. digitifera* and *Acropora* sp. 1, using short read mapping data from the *Acropora* sp.1 sample (ID: Asp1S2003) with the highest short read coverage, a consensus sequence of the *WDR59* coding region was extracted. The consensus sequence is shown in the supplemental material. The evolutionary divergence between *WDR59* sequence of *A. digitifera* (adig_s0048.g28.t1) and *Acropora* sp. 1 (extracted consensus sequence) estimated by MEGA 7 [40] was 0.0045.

A phylogenetic tree using the consensus sequence of *Acropora* sp. 1 and the *WDR59* sequence of 14 other *Acropora* species showed that *WDR59 of Acropora* sp. 1 and *A. digitifera* formed a monophyletic clade (Fig. S2).

To determine whether three amino acid differences in *WDR59* between *A. digitifera* and *Acropora* sp. 1 are shared with other species or are specific to *Acropora* sp. 1, we analyzed *WDR59* in 15 *Acropora* species (Table S7). First, we aligned the WDR59 sequence of 14 *Acropora* species (excluding *A. cytherea* due to a possible partial sequence) with that of *A. digitifera* and *Acropora* sp. 1 (Fig. S3) and found that one of the three amino acid changes (adig_s0048.g28.t1: CDS; 2239 C > T, amino acid sequence; Pro747Ser) is specific to *Acropora* sp. 1 (Fig. S3).

Next, we manually checked mapping reads around *WDR59* and found that *Acropora* sp. 1 colonies have a 24 bp deletion located in exonic sequence 38 bp downstream of the *Acropora* sp. 1-specific amino acid change. To verify this deletion, we amplified and sequenced the region containing the deletion by PCR from genomic DNAs of *A. digitifera* (*n* = 7) and *Acropora* sp. 1 (*n* = 14). We confirmed the deletion and found two additional amino acid differences between *A. digitifera* and *Acropora* sp. 1, upstream (15 bp) and downstream (14 bp) of the 24 bp deletion (Fig. S4). These two additional mutations found in Sanger sequencing were excluded from SNV analysis during the variation filtering process due to low mapping coverage (DP > 3) of short reads. Among the differences between *A. digitifera* and *Acropora* sp. 1, two amino acid changes and a deletion are shared with *A. nasuta*, and one amino acid change is specific to *Acropora* sp. 1 (Figs. S5 and S6).

To estimate the position of the amino acid change specific to *Acropora.* sp. 1, we used Phyre2 [53] to search for proteins highly similar to *A. digitifera WDR59* in known structure databases. As a result, *S. cerevisiae* Sea3, the yeast counterpart of mammalian WDR59, was highly similar to *A. digitifera WDR59* (E-value = 0, Identity = 29%). *S. cerevisiae* Sea3 (WDR59) has an α-solenoid interface region where Sea3 (WDR59) interacts with the other subunit to form a complex, Sea2 (WDR24) [54]. The α-solenoid interface region is located from amino acids 782 to 1,061 of *S. cerevisiae* Sea3 (WDR59) [54]. An alignment of *A. digitifera* WDR59 with *S. cerevisiae* Sea3 (*WDR59*) (Fig. S7) showed that the amino acid changes specific to *Acropora* sp. 1 are located in the α-solenoid interface region.

To check expression of candidate genes during spawning, we analyzed two RNA-seq datasets from published studies: *A. digitifera* [42] and *A. tenuis* [43]. The expression values (TPM) of *WDR59* are shown in Figures S8-S9 and Tables S9-S10. WDR59 expression level (TPM) in *A. digitifera* were TPM: 24.4–30.1(rank percentile of TPM 30–39%) and WDR59 expression level (TPM) in *A. tenuis* were TPM: 29.6–42.5 (rank percentile of TPM 20.5–30.4%) during spawning or reproductive seasons.

## Discussion

### *A. digitifera* and *Acropora* sp. 1 are useful for understanding timing of gametogenesis in *Acropora*

Studying the timing of gamete maturation in corals using a population genetic approach, as in this study, provides insights into genetic mechanisms of coral gametogenesis and speciation in corals. Therefore, we propose *A. digitifera* and *Acropora* sp. 1 as a model species pair for studying mechanisms of spawning month determination and speciation in corals.

One of the advantages of using these two species is their clear phenotypic difference in timing of spawning. In Okinawa, *A. digitifera* spawns in May or June, whereas *Acropora* sp. 1 spawns in August [23–25]. Continuous observations of oocyte volume revealed that gamete maturation is later in *Acropora* sp. 1 than in *A. digitifera* [23]. The difference in gamete maturation is expected to lead to reproductive isolation. Indeed, PCA showed clear genetic differentiation between the two species, even though their overall genetic distance is low.Therefore, gene flow between *A. digitifera* and *Acropora* sp. 1 is limited, which is considered an initial stage of speciation. This is consistent with the phylogenetic analysis showing that while bootstrap support is 100 for the respective monophyletic groups of *A. digitifera* and *Acropora* sp. 1, site concordance is low.

The low genetic divergence between *A. digitifera* and *Acropora* sp. 1 is another advantage in studying genes responsible for spawning timing mechanisms and speciation. Genomic divergence between these two species is low (F_ST_ = 0.10225), consistent with a previous microsatellite marker study [25]. Using this low-genomic diverged species pair, we identified 34 HDRs containing 60 genes. These genomic regions and candidate genes may be responsible for morphological and ecological differences between the two species. Among the 60 candidate genes, 6 genes possessed putative orthologs broadly distributed from yeast to humans (Table 1), suggesting their potential involvement in fundamental biological processes essential for life. These evolutionarily conserved genes may contribute to the phenotypic differences observed between *A. digitifera* and *Acropora* sp. 1, despite their ancient origins and functional conservation across diverse taxa. In contrast, approximately half (48%) of the candidate genes lacked detectable orthologs in the four model organisms employed for ortholog prediction in this study (Table S4). This finding suggests that these genes may be involved in functions specific to cnidarians or corals. Further analyses of gene expression differences in different months, functional changes resulting from highly diverged SNVs are expected to advance research on the mechanism of spawning month determination and speciation in corals.

### Genes that may determine morphological differences between two species

Morphological characteristics of *Acropora* sp. 1 include shorter branches and a flatter colony shape than *A. digitifera* [23, 24]. These morphological differences reflect differences in skeletal form [55]. The alpha collagen-like proteins are skeletal organic matrix proteins involved in skeletal formation in *Stylophora pistillat*a [56, 57] and *A. millepora* [50]. In this study, we identified one gene (Gene ID: adig_s0171.g21) which is homolog of *A. millepora* collagen alpha-1(I) chain-like (LOC114955150) in HDRs, and these genes are likely responsible for species-specific differences in skeletal morphology.

### mTORC1 may contribute to gametogenesis of *A. digitifera*

As far as we could confirm among the 15 *Acropora* coral species for which we obtained putative orthologs of WDR59 and could verify their sites, amino acid changes (adig_s0048.g28.t1: CDS; 2239 C > T, amino acid sequence; Pro747Ser) were specific to *Acropora* sp. 1 (Fig. S3). Among the 15 *Acropora* coral species aligned in Fig. S3, previous studies confirmed through observations at Akajima, Okinawa [21] that *A. cytherea* (May), *A. florida* (June), *A. gemmifera* (May-June), *A. hyacinthus* (May-June), *A. microphthalma* (May), *A. millepora* (May), *A. nasuta* (May), and *A. tenuis* (May) spawn during the typical synchronized spawning period (May-June). This suggests that the mutation specific to *Acropora* sp. 1 may possibly associate with reproductive timing differences.

**Fig. 4:**
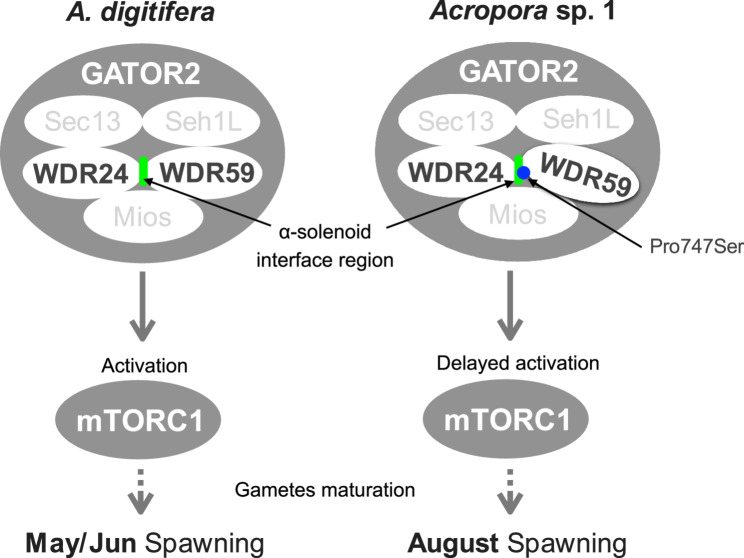
Schematic representation of a hypothesis proposed in this study. Regulation of mTORC1 by GATOR2 and components of GATOR2 is based on previous studies [51, 52, 68]. An *Acropora* sp. 1-specific mutation in the WDR59/WDR24 interaction region is indicated with a blue circle

WDR59 is one of the components of a mechanistic target-of-rapamycin complex 1 (mTORC1) activator, GATOR2 [51, 58] (Fig. 4). mTORC1 is one of the components of mTOR signaling pathway involved in meiotic entry and gametogenesis as well as nutrient sensing [59]. The mTOR signaling pathway is present in *A. digitifera* as shown in KEGG (https://www.kegg.jp/pathway/adf04150). Regulation of meiotic entry by mTORC1 is conserved from yeast to mammals. Downregulation of mTORC1 activity promotes the transition from mitotic to meiotic cycles in *Saccharomyces cerevisiae*, *Schizosaccharomyces pombe* [60, 61], and *Drosophila* [52]. In mice, mTORC1 is required for spermatogonial differentiation [62] and oogenesis [63]. Activated mTORC1 drives oocyte development and growth in *Drosophila* oogenesis [64].In Cnidarians, for role of mTORC1, Voss et al. 2022 have demonstrated that nutrient sensing through mTOR signaling is essential for symbiosis with Symbiodiniaceae in both the endosymbiosis model Aiptasia and *A. tenuis* [65]. To the best of our knowledge, the function of mTORC1 in gametogenesis among Cnidarians has been little discussed. One exception is a study about the kinase, Mos, which regulates oocyte maturation in the jellyfish, *Clytia hemisphaerica* [66]. Treatment of oocytes with rapamycin, a potent inhibitor of mTORC1, suggested that the mTORC1 signaling pathway controls one *Mos* paralog translation during oocyte growth [66]. Moreover, in *Hydra oligactis*, continuous exposure to rapamycin results in fewer mature sperm cells than in untreated individuals [67]. Hence, mTORC1 is likely associated with gametogenesis in cnidarians, including *Acropora* species.

The *Acropora* sp. 1-specific amino acid change in WDR59 is located in a region where WDR59 interacts with one of the other GATOR2 components to form the complex (GATOR2). This amino acid change may cause slight differences in stability or structure of GATOR2 through affinity of WDR59 with its counterpart. Our analysis of RNA-seq data from previous studies [42, 43] indicated and the relatively high expression of *WDR59* in *A. digitifera* (TPM: 24.4–30.1, rank percentile of TPM 30–39% among all genes in the sample) and *A. tenuis* (TPM: 29.6–42.5, rank percentile of TPM 20.5–30.4% among all genes in the sample) during spawning or reproductive seasons. WDR59 is one of the components of GATOR2, the observed relatively high expression of *WDR59* indicates that GATOR2 has a possible role in reproduction of *Acropora*. The RNA-seq data did not suggest that the expression level of the *WDR59* may change during or before or after spawning. The absence of significant transcriptional changes in *WDR59* during spawning periods does not necessarily indicate its lack of involvement in gametogenesis. If *WDR59* is indeed involved in gametogenesis, it would likely function through post-translational mechanisms rather than transcriptional regulation. This hypothesis is consistent with the known mechanism by which GATOR2, which contains WDR59 as a component of the complex, regulates mTORC1 activity through post-translational modifications [59, 69] The RNA-seq data from the previous studies [42, 43] we analyzed were derived from RNAs extracted from coral branch fragments. We cannot rule out the possibility that changes in *WDR59* expression during gametogenesis could be observed by analyzing reproductive tissue-specific RNA-seq.

In *Drosophila* oogenesis, GATOR2 activates mTORC1, and active mTORC1 is required to start oocyte development [52]. Since regulation of gametogenesis by mTORC1 has been reported in *Drosophila*, meiotic entry and oocyte development in *Acropora* species is also likely controlled by mTORC1 activity, regulated by GATOR2. In other words, the difference in timing of gamete maturation between *A. digitifera* and *Acropora* sp. 1 [23] could potentially be caused by an amino acid substitution in WDR59 that slightly affects timing of mTORC1 activation via GATOR2 though we did not analyzed the expression of *WDR59* in *Acropora* sp. 1, and possible involvement of the *WDR59* in the regulation of gametogenesis remains speculative. Coral gametogenesis, particularly oogenesis, depends on energy supply from Symbiodiniaceae [19], and the mTOR signaling pathway has been reported to be involved in symbiosis with Symbiodiniaceae [65]. Based on these findings, if we assume that *WDR59* mutations affect the mTOR signaling pathway, we cannot exclude the possibility that *WDR59* mutations influence nutrient transport with Symbiodiniaceae, which in turn affects gametogenesis, particularly oogenesis.　Note that even though we focused on *WDR59* in this study, a combination of genetic factors, including genes in other HDRs, may be responsible for differences in spawning timing.

## Conclusion

In this study, we analyzed genomes of two closely related *Acropora* species with different spawning months, May/June and August. Our analyses revealed that 60 genes are genetically diverged between the two species. One of these is a component of mTORC1 activator, suggesting that this gene may be associated with the difference in spawning times of these two species. Since the phylum Cnidaria, including corals, is located in the basal lineage of the animal kingdom, studies revealing the function of mTORC1 in gametogenesis in corals will provide insights into evolution of gametogenesis regulation. Future studies of the two coral species used in this study will shed light on mechanisms that determine the timing of coral spawning.

## Supplementary Information

Supplementary Material 1

Supplementary Material 2

## Data Availability

The data sequenced in this study were deposited in the DNA Data Bank of Japan (DDBJ) Sequenced Read Archive under accession numbers DRR420947-DRR420962. Nucleotide sequences for identification of a deletion in WDR59 is provided in the Supplementary Material. The accession numbers to the respective data analyzed in this study are listed in Table S1.
