# Supplementary material for: Comparative genomics of two closely related Acropora coral species with different spawning seasons reveals genomic regions possibly associated with gametogenesis
Source: BMC Ecol Evol. 2025 Sep 1;25:88. doi: 10.1186/s12862-025-02432-5 (PMC12400658; doi:10.1186/s12862-025-02432-5)
Supplement: Supplementary file 2 — Supplementary Material 2 [file 12862_2025_2432_MOESM2_ESM.docx]

Supplementary Material

Contents

1 Materials and Methods

1.1 Samples

1.1.1 Sample collection

1.1.2 DNA Extraction and Sequencing

1.2 Alignment and variant calling of *Acropora digitifera* and *Acropora* sp.1

1.2.1 Short reads alignment

1.2.2 Variant calling

1.2.3 Variant Filtering

1.3 Alignment and variant calling of 15 other Acropora species

1.4 SNV analysis

1.4.1 Phylogenetic analysis

1.4.2 PCA analysis

1.4.3 Genome scan of highly diverged regions

1.4.4 Genes in highly diverged regions (HDRs)

1.5 Variation of WDR59

1.5.1 Identification of a deletion in WDR59 among *Acropora* sp.1

1.5.2 Identification and alignment of WDR59 in other *Acropora* species

1.5.3 Extraction of the consensus sequence of *WDR59* from *Acropora* sp. 1

1.5.4 Phylogenetic three construction of the *WDR59* in *Acropora* species

1.5.5 Gene expression of the *WDR59*

2 Supplemental Figures

3 Supplemental Tables

4 Supplemental Data

4.1 Nucleotide sequences for identification of a deletion in WDR59

4.2 Consensus sequence of *WDR59* from *Acropora* sp. 1

**1 Materials and Methods**

**1.1 Samples**

**1.1.1 Sample collection**

In total, 16 samples from 16 *Acropora* sp.1 colonies were collected during 2018-2020 in Okinawa, Japan, under permission of the Aquaculture Agency of Okinawa Prefecture (permit numbers 30-29, 31-43, and 31-68). Dr. Akira Iguchi identified *Acropora* sp.1 colonies based on their morphology.

On 30 July 2018, one *Acropora* sp.1 (Colony ID: Asp1_c) colony was collected from Bise-zaki and maintained in an aquarium at the Sesoko Station, Tropical Biosphere Research Center, University of the Ryukyus. We observed spawning of this colony on 5 August, and sperm was collected from this colony and stored at -80.

On 3 August 2019, we collected seven colonies (Colony IDs: Asp1B1901- Asp1B1907) from Bise-zaki, and a branch fragment was collected and preserved in RNAlater (Waltham, MA USA) from each of seven colonies. We maintained these colonies in an aquarium at the Sesoko Station for spawning observations.

At the beginning of August 2020, we collected eight colonies (individual IDs: Asp1S2001- Asp1S2008) from the Sesoko-jima reef. A branch fragment was collected and preserved in RNAlater (Waltham, MA, USA) from each of the seven colonies.

**1.1.2 DNA Extraction and Sequencing**

We extracted genomic DNA from *Acropora* sp.1 colonies for the genome DNA library. We used DNeasy Plant Mini Kits (QIAGEN, Hilden, Germany) for DNA extraction from 15 branch fragments. For DNA extraction from the sperm sample, we used DNeasy Blood & Tissue Kits (QIAGEN, Hilden, Germany). Following the manufacturer's instructions, we constructed DNA libraries of 16 samples using an NEBNext Ultra II DNA Library Prep Kit (New England Biolabs, Ipswich, MA, USA) and NEBNext Multiplex Oligos for Illumina 96 Unique Dual Index Primer Pairs (New England Biolabs). The 15 libraries from branch tissues were sequenced on an Illumina HiSeqX Ten, and one library from sperm was sequenced on an Illumina HiSeq 2500.

**1.2 Alignment and variant calling of *Acropora digitifera* and *Acropora* sp.1**

**1.2.1 Short-read alignment**

We used short-reads of 11 *A. digitifera* colonies and 14 *Acropora* sp. 1 colonies. In addition to our sequencing data from *Acropora* sp.1, we downloaded complete genome sequences of 11 colonies of *A. digitifera* from the DNA Data Bank of Japan (DDBJ) (Table S1). Raw 150 bp paired-end Illumina short reads saved as FASTQ files were trimmed with fastp (Chen, et al. 2018) to remove low-quality reads and Illumina adapters using modified parameters (-l 50 -q 30).

### Trimming Reads (example)

fastp --in1 read_1.fastq.gz --in2 read_2.fastq.gz --out1 read.R1.trimmed.fastq.gz --out2 read.R2.trimmed.fastq.gz -l 50 -q 30

Trimmed reads were then aligned to the *A. digitifera* genome assembly ver. 2.0 (<https://marinegenomics.oist.jp/adig/viewer/download?project_id=87>) (Shinzato, et al. 2021) with bowtie2 version 2.3.3.1 (Langmead and Salzberg 2012) using modified parameters (--score-min L,0,-0.2). Alignment results were saved as Sequence Alignment/Map (SAM) format files and converted to Binary Alignment/Map (BAM) format files using samtools version 1.3.1(Danecek, et al. 2021). If the same sample-derived reads are split into multiple FASTQ files, each read file is aligned separately, and the output BAM files are sorted, indexed, and concatenated for each sample with samtools version 1.3.1 (Danecek, et al. 2021). After removing nonspecific reads from bam files by samtools version 1.3.1, read coverage was checked using goleft version 0.2.4 (https://github.com/brentp/goleft). Individual information (read group) was added to each of the BAM files by GATK version 4.1.6.0 (McKenna, et al. 2010).

### Map reads (example)

bowtie2 -q -x GCA_014634065.1_Adig_2.0 -1 read.R1.trimmed.fastq -2 read.R2.trimmed.fastq FILE.sam -p 4 --score-min L,0,-0.2 -S

### Convert SAM to BAM (example)

samtools view -bS -h FILE.sam > FILE.bam

### Merge BAM files (example)

samtools merge FILE.merged.bam FILE1.bam FILE 2.bam

### Filter MAPQ2 (example)

samtools view -q 2 -b 1_ FILE.merged.bam > FILE.merged.q2.bam

### Coverage

goleft covstats FILE.merged.q2.bam > FILE.merged.q2.coverage.txt

### Add read group (example)

gatk AddOrReplaceReadGroups -I FILE.merged.q2.bam -O FILE.merged.q2.new.bam -PL ILLUMINA -LB lib1 -PU unit1 -SM FILE

### Sort BAM

samtools sort FILE.merged.q2.new.bam > FILE.merged.q2.new.sort.bam

### Add index to BAM (example)

samtools index FILE.merged.q2.new.sort.bam

### Mark Duplicates (example)

Gatk MarkDuplicates -I FILE.merged.q2.new.sort.bam -M FILE_marked_dup_metrics.txt -O FILE.merged.q2.new.sort.md.bam

### Add index to BAM (example)

samtools index FILE.merged.q2.new.sort.md.bam

**1.2.2 Variant calling**

The HaplotypeCaller program, which is provided in GATK version 4.1.6.0 (McKenna, et al. 2010) was used to call genetic variants in each sample. Called variants from each sample were saved as individual files (called gvcf files), and individual files were combined using GATK CombineGvcf program. GATK GenotypeGVCFs program was used to call genotypes among all combined samples, and genotyped variants were saved as one file (called "vcf file").

### Haplotype Calling (example)

gatk HaplotypeCaller -R REFERENCE.fasta -I FILE.q2.new.sort.md.bam -ERC GVCF -output FILE.q2.raw.g.vcf.gz

### genotypeGVCF (example)

gatk GenotypeGVCFs -R REFERENCE.fa -V gvcf -O vcf

**1.2.3 Variant Filtering**

Following GATK "best practices" (https://gatk.broadinstitute.org/hc/en-us/articles/360035890471) (DePristo, et al. 2011; Poplin, et al. 2017), hard-filtering with minor modifications was applied to genotyped variants. For genotype variants, only the threshold for low mapping quality (MQ) was changed from 40 to 20, while other thresholds were left at default settings. We excluded sites with low QUAL score normalized by allele depth (QD < 2.0); low QUAL score (QUAL < 30.0); high strand odds ratio **(**SOR > 3.0); high Fisher strand value (FS > 60.0); low mapping quality value (MQ < 20.0); low mapping quality rank sum score (MQRankSum < -12.5); low read position rank sum score (ReadPosRankSum < -8.0).

After GATK hard-filtering, we excluded sites with missing sites and indels, low minor allele frequency (maf < 0.05) to remove doubletone, and low depth values (minDP < 3) using VCFtools version 0.1.16 (Danecek, et al. 2011). We further selected biallelic SNVs with bcftools version 1.9 (Danecek, et al. 2021).

To remove SNVs, a cutoff value for maxDP was set for each vcf file, and sites with DP values higher than twice the average DP of the individual's biallelic SNVs were excluded from the analysis. First, vcf files for each individual were extracted from the combined vcf files with VCFtools version 0.1.16 (Danecek, et al. 2011). For each individual vcf file, the following operations were performed. The vcftools option, “site-depth”, was used to extract the depth of each site, the average DP was calculated, and a bed file was created showing sites where the DP value was higher than twice the average DP of the bi-allelic SNV for that individual. All bed files for each individual were combined, and duplicate entries in the combined bed files were deleted. Bi-allelic SNVs were selected from the combined vcf, excluding the region specified in the bed file.

To remove SNVs that were not under Hardy-Weinberg equilibrium we checked the p-value for each site from a Hardy-Weinberg Equilibrium test (Chiu, et al. 2020) using VCFtools version 0.1.16 (Danecek, et al. 2011). First, the maxDP-filtered vcf file was split into two vcf files for each of the two species. The vcf file for each species was used to determine a p-value for each site from the Hardy-Weinberg Equilibrium test. Only sites with a p-value < 0.05 were used to perform PCA without out-groups (explained in 1.4.2 PCA) and calculate F_ST_ s (explained in 1.4 .3 Genome scan of the highly diverged region)*.*

### Variant filtration (example)

gatk VariantFiltration -R ref_fasta -V in_vcf -O out_name_hardfilterMQ20.vcf --filter-expression "QD < 2.0" --filter-name "QD2" --filter-expression "QUAL < 30.0" --filter-name "QUAL30" --filter-expression "SOR > 3.0" --filter-name "SOR3" --filter-expression "FS > 60.0" --filter-name "FS60" --filter-expression "MQ < 20.0" --filter-name "MQ20" --filter-expression "MQRankSum < -12.5" --filter-name "MQRankSum-12.5" --filter-expression "ReadPosRankSum < -8.0" --filter-name "ReadPosRankSum-8"

### SelectVariants only pass (example)

gatk SelectVariants -R ref_fasta -V out_name_hardfilterMQ20.vcf -O out_name_hardfilterMQ20_pass.vcf --exclude-filtered true

### Filtering with vcftools (example)

vcftools --gzvcf in_vcfgz --remove-indels –maf 0.05 --max-missing 1 --min-meanDP 3 –minDP 3 --recode --recode-INFO-all --out out_name

### Select biallelic sits with bcftools (example)

bcftools view -Ob -o temp.bcf out_name.recode.vcf.gz

bcftools index -f temp.bcf

bcftools view -m2 -M2 temp.bcf -Oz -o out_vcfgz

### Make individual vcf (example)

vcftools --gzvcf out_vcfgz --indv NAME --recode --recode-INFO-all --out NAME

bgzip -c NAME.recode.vcf > NAME.recode.vcf.gz

rm NAME.recode.vcf

vcftools --gzvcf NAME.recode.vcf.gz --site-depth --out NAME

### R

### Load tidyverse package
library(tidyverse)

### Import "samplename.ldepth"

ind_depth <- read_delim("samplename.ldepth", delim = "\t", col_names = c("CHROM", "POS", "SUM_DEPTH","SUMSQ_DEPTH"), skip = 1)

### MeanDP

b <-mean(ind_depth$SUM_DEPTH)

### Excluding decimal points

c <- round(b, digits=0)

### output the result

df <- data.frame(name = c("samplename"), meanDP = c(c))

write.table (df,"samplename.meanDP.txt",append=T, quote=F, col.names=F)

### Merge meanDP results

cat *.meanDP.txt > merged_meanDP.txt

cat merged_meanDP.txt | awk '｛print $2"\t"$3｝' > merged_meanDP_modified.txt

#!/usr/bin/bash

work_dir=" "

list="merged_meanDP_modified.txt"

### Specify the third column where DEPTH is listed in a file

column=3

### Specify how many times the DP cutoff value is to be multiplied by the meanDP value

number=2

set -e

NAME=`basename $0`

LOGFILE=$work_dir/$NAME_log.txt

{

date

mkdir -p cutoff_meanDPx$number

cat $list | while read line

do

sample="$(echo "$line" | cut -f1 -d' ')"

depth="$(echo "$line" | cut -f2 -d' ')"

maxDP=`expr $depth \* $number`

echo "$sample $depth $maxDP"

awk -v col=$column -v DP=$maxDP -F "\t" '$col>DP {print $1 "\t" $2-1 "\t" $2}' $sample.ldepth > cutoff_meanDPx$number/$sample.bed

done

cd cutoff_meanDPx$number

cat *.bed > merged.bed.tmp

sort merged.bed.tmp | uniq > remove_list_meanDPx$number.bed

rm merged.bed.tmp

echo "end"

} >> "$LOGFILE" 2>&1

### Output a vcf file excluding the sits specified by the bed file

bedtools intersect -v -a in_vcf -b in_bed -wa -header > out_vcf

### Remove SNVs that were not under Hardy-Weinberg equilibrium

#!/usr/bin/bash

work_dir1=""

in_vcf=" "

out_vcf=""

pop1="Adig"

pop2="Asp1"

set -e

NAME=`basename $0`

LOGFILE= $ { work _ dir1 } / ${NAME}_log.txt

{

date

cd $work_dir1

vcftools --vcf $in_vcf --indv AdigS1601 --indv AdigS1602 --indv AdigS1603 --indv AdigS1604 --indv AdigS1606 --indv AdigS1607 --indv AdigS1610 --indv AdigShinzato2011 --indv Adig_Shinzato2015_KrE6 --indv Adig_Shinzato2015_KrE8 --indv AdigiShinzato2020 --recode --recode-INFO-all --out $pop1

bgzip $pop1.recode.vcf

vcftools --gzvcf $pop1.recode.vcf.gz --hardy --out $pop1

cat $pop1.hwe | awk -F "\t" '$6<0.05 {print $1 "\t" $2-1 "\t" $2}'> $pop1.p005.bed

vcftools --vcf $in_vcf --indv Asp1B1901 --indv Asp1B1902 --indv Asp1B1903 --indv Asp1B1904 --indv Asp1B1905 --indv Asp1B1906 --indv Asp1B1907 --indv Asp1S2001 --indv Asp1S2002 --indv Asp1S2003 --indv Asp1S2005 --indv Asp1S2007 --indv Asp1S2008 --indv Asp1c --recode --recode-INFO-all --out $pop2

bgzip $pop2.recode.vcf

vcftools --gzvcf $pop2.recode.vcf.gz --hardy --out $pop2

cat $pop2.hwe | awk -F "\t" '$6<0.05 {print $1 "\t" $2-1 "\t" $2}'> $pop2.p005.bed

cat *.bed > merged.bed.tmp

sort merged.bed.tmp | uniq > remove_list_HWEp005.bed

rm merged.bed.tmp

bedtools intersect -v -a $in_vcf -b remove_list_HWEp005.bed -wa -header > $out_vcf

} >> "$LOGFILE" 2>&1

**1.3 Alignment and variant calling of 15 other *Acropora* species**

We downloaded genome sequences of 15 other *Acropora* species from DDBJ. Downloaded raw reads were trimmed in the same manner as described above (1.2.1 Short reads alignment). Trimmed reads were then aligned to the *A. digitifera* genome assembly ver. 2.0 (Shinzato, et al. 2021) with bowtie2 ver. 2.3.3.1 (Langmead and Salzberg 2012) using the default setting. A gvcf file containing genotyped variants was generated for each of the 15 species and combined with the *A. digitifera* and *Acropora* sp.1 gvcf files. We genotyped variants and filtered variants in the same manner described above (1.2.2 Variant calling and 1.2.3 Variant Filtering) with minor changes. The low minor allele frequency(maf) was 0.03 to remove doubleton, and the Hardy-Weinberg Equilibrium test was not performed.

**1.4 SNV analysis**

**1.4.1 Phylogenetic analysis**

For phylogenetic analysis using IQ-TREE, we initially used 885,405 SNVs data from 17 Acropora species: 14 individuals for *Acropora* sp. 1, 11 individuals for A. digitifera, and one individual each for the remaining 15 Acropora species. Since sites where no individuals have homozygous variants become invariant sites in IQ-TREE phylogenetic analysis, IQ-TREE automatically filtered the data to 798,399 variable sites, which were then used for the actual phylogenetic analysis.For the IQ-TREE analysis, we used the GTR+ASC model. The ASC (ascertainment bias correction) model is specifically designed for SNP data where invariant sites are excluded, as it corrects for the bias introduced by using only variable sites. The GTR (General Time Reversible) model allows for different rates of nucleotide substitution between all pairs of nucleotides. We performed bootstrap analysis with 1,000 replicates to assess branch support and conducted site concordance analysis with 100 replicates to evaluate the concordance of individual sites with the inferred phylogeny.

# Extract variable sites for analysis

iqtree3 -s input.vcf.phy -m GTR+ASC

# Create phylogenetic tree

iqtree3 -s input.vcf.phy.varsites.phy -nt AUTO -m GTR+ASC -B 1000

# Perform site concordance analysis using the generated tree file

iqtree3 -te input.vcf.phy.varsites.phy.treefile -s input.vcf.phy.varsites.phy --scfl 100 --prefix concord -m GTR+ASC

**1.4.2 PCA analysis**

To show the separation of *A. digitifera* and *Acropora* sp. 1, we performed principal components analysis (PCA) on the genome-wide pruned 72,051 SNVs using PLINK v1.90 (http://pngu.mgh.harvard.edu/purcell/plink/) using a vcf file including 11 *A. digitifera* colonies, 14 *Acropora* sp. 1colonies described above (1.2.2 Variant calling and 1.2.3 Variant Filtering).

**1.4 .3 Genome scan of highly diverged region*s (HDRs)***

We calculated F_ST_ s (Hudson, et al. 1992) for 10-kb windows with 1-kb increments along each scaffold (>10 kb) using a sliding-window approach by PopGenome (Pfeifer, et al. 2014). First, we extracted a 10-kb window containing the top 0.1% of the F_ST_ distribution and created a bed file showing the genomic positions of the top 0.1% windows by manually. Using the bed file, we extracted information on SNVs in the top 0.1% windows and genotype information for each sample using bcftools version 1.9 (Danecek, et al. 2021). Based on the genotype information, we selected SNVs for which the allele is fixed in one population and for which there was no homozygote for the allele in the other population. We considered these SNVs as diverged SNVs. Among the top 0.1% of windows, we extracted windows with diverged SNVs and merged the overlapping regions. These combined regions were considered highly diverged regions (HDRs).

### R

setwd("")

library(PopGenome)

list <- read.table("vcf_scaf_len.list")

len <- nrow(list)

for ( i in 1:len) {

chr_name <- list[i,1]

chr_len <- list[i,2]

GENOME.class <- readVCF("Adig_sp1_nph3_hardfilter_MQ20maxDPx2HWE.vcf.gz", numcols=10.000, tid=paste(chr_name), from=1, to=chr_len, approx=FALSE, out="", parallel=FALSE, gffpath=FALSE)

Adig <- c("AdigS1601","AdigS1602","AdigS1603","AdigS1604","AdigS1606","AdigS1607","AdigS1610","AdigShinzato2011","Adig_Shinzato2015_KrE6","Adig_Shinzato2015_KrE8","AdigiShinzato2020")

Asp1 <- c("Asp1B1901","Asp1B1902","Asp1B1903","Asp1B1904","Asp1B1905","Asp1B1906","Asp1B1907","Asp1S2001","Asp1S2002","Asp1S2003","Asp1S2005","Asp1S2007","Asp1S2008","Asp1c")

populations <- list(Adig, Asp1)

GENOME.class <- set.populations(GENOME.class, populations, diploid=TRUE)

###split the data in 10kb windows

GENOME.class.slide <- sliding.window.transform(GENOME.class, width=10000, jump=1000, type=2)

###To convert those windows into a genomic numeric position

genome.pos <- sapply(GENOME.class.slide@region.names, function(x){

split <- strsplit(x," ")[[1]][c(1,3)]

val <- mean(as.numeric(split))

return(val)

})

###total number of windows

windows <- length(GENOME.class.slide@region.names)

###FST

GENOME.class.slide <- F_ST.stats(GENOME.class.slide, mode="nucleotide")

###pairwise nucleotide FST

pairwise.FST <- t(GENOME.class.slide@nuc.F_ST.pairwise)

fst_result <- paste(chr_name,genome.pos,GENOME.class.slide@region.names,pairwise.FST)

write.table(fst_result,paste("FstResult_",chr_name,".txt",sep = ""),quote=F)

}

### extract genotype information

bcftools view in_vcfgz -R in_bed -o name.extract.vcf

bcftools query -f '%CHROM\t%POS\t%REF\t%ALT[\t%GT]\n' name.extract.vcf > name.extract.vcf_GT.txt

**1.4 .4 Genes in highly diverged regions**

We considered genes in HDRs as candidate genes related to differences between the two species. To identify functional annotations of these genes, we searched putative orthologs of 60 candidate genes using OrthoFinder(Emms and Kelly 2019). we performed ortholog inference using OrthoFinder (Emms and Kelly 2019) with transcripts from *A. digitifera* and the following four model organisms: *Saccharomyces cerevisiae*, *Drosophila melanogaster*, *Danio rerio*, and *Homo sapiens.*

orthofinder -f proteome -t 4 -a 4 -S diamond_ultra_sens -M msa -A muscle -T fasttree -og -y

We used PANTHER (https://pantherdb.org) to search for the annotation orthologous genes corresponding to each *A. digitifera* candidate gene in model organisms and compiled the ortholog information (PANTHER Family/Subfamily) from the model organisms. For genes for which orthologs were not identified in any of the four model organisms, we searched homologous genes in the National Center for Biotechnology Information (NCBI) nucleotide database by Blast search （https://blast.ncbi.nlm.nih.gov/Blast.cgi）at default settings (Altschul, et al. 1990). The top hits (with *e* ≥ 1e^–30^ and identity ≥ 90% were regarded as homologous genes.

We determined whether diverged SNVs cause amino acid changes using the Amino Acid Changes annotation tool in CLC Genomics Workbench 11.0 (QIAGEN, Aarhus, Denmark). We used the *A. digitifera* genome version 2 as a reference track and a bed file including the location of diverged SNVs (explained in section 1.4.3 Genome scan of highly diverged regions). We selected genetic code = standard.

**1.5 Variation of WDR59**

1.5.1 Identification of a deletion in *WDR59* among *Acropora* sp.1

The presence of one deletion in the WDR59 gene among *Acropora*. sp. 1 was confirmed by visual inspection of mapping results. To identify the deletion, we amplified the genomic region containing the expected deletion using PCR with PrimeSTAR GXL DNA Polymerase (Takara, Shiga, Japan) and the following primers: 5’-CTCCATATTCTAACATCTCTG-3' and 5'- AAAACGTAGCTTGCTAAAGC-3'. PCR was performed using GeneAmp PCR System 9700 (Applied Biosystems, Carlsbad, CA, USA) with the following conditions: denaturation for 1 min 30 sec at 93 °C, followed by 30 cycles of denaturation for 30 sec at 93 °C, annealing for 30 sec at 55 °C, and extension for 30 sec at 72 °C. We used the following genomic DNAs as templates for PCR: Genomic DNAs extracted from 7 *A. digitifera* colonies (sample ID: AdigS1601–4, AdigS1606–07 and AdigS1610) and 14 Acropora sp. 1 colonies (sample ID: Asp1B1901-07, Asp1c, Asp1S2001-03, Asp1S2005, and Asp1S2007-08). We determined the sequences of PCR products using a Genetic Analyzer 3500 (Thermo Fisher Scientific) with the same primers used for PCR. All determined sequences were aligned with the reference sequence (*A. digitifera* scaffold: sc0000048_arrow_pilon) using ClustalW with default parameters in MEGA ver. 7 (Kumar, et al. 2016).The result is shown in Figure S4.

**1.5.2 Identification and alignment of WDR59 in other *Acropora* species**

The genome assemblies of 14 *Acropora* species (*A. tenuis, A.yongei, A. intermedia, A. gemmifera, A. awi, A. florida, A. selago, A. hyacinthus, A. cytherea, A. muricata, A. echinata, A. acuminata, A. nasuta, and A. microphthalma*) were downloaded from the OIST Marine Genomics Unit Genome Browser (https://marinegenomics.oist.jp/gallery). FASTA files containing all amino acid sequences for each species were extracted from whole genome sequences using gffread with GTF file information as input. The extracted amino acid sequence FASTA files contained sequences that were not correctly translated, and such sequences cannot be handled by OrthoFinder, so they were removed using command-line operations. The sequence removal rate was 0.6-0.8% (Table S6).

# Extract amino acid sequences from GTF and genome FASTA

gffread -g genome.fasta -y proteins.fasta input.gtf

# Remove line breaks from sequences

seqkit seq -w 0 proteins.fasta > proteins2.fasta

# Create a list of sequence names containing "." in the sequence

awk '/^>/ {header=$0; getline; if(/\./) print header}' proteins2.fasta > proteins2_remove_list.txt

# Read the sequence name list from file and extract sequences not included in the list

seqkit grep -f proteins2_remove_list.txt proteins2.fasta > input_clean.fa

To find the orthologs of 60 candidate genes in *Acropora* species, putative orthologous genes of *WDR59* and *WDR24* were searched in the reference transcriptome of each of the 14 *Acropora* species using OrthoFinder version 2.5.4(Emms and Kelly 2019). with following command.

orthofinder -f proteome -t 4 -a 4 -S diamond_ultra_sens -M msa -A muscle -T fasttree -og -y

**1.5.3 Extraction of the consensus sequence of *WDR59* from *Acropora* sp. 1**

To confirm the similarity of *WDR59* between *A. digitifera* and *Acropora* sp. 1, we extracted a consensus sequence for CDS of WDR59 from short read mapping data of an *Acropora* sp.1 sample (ID: Asp1S2003) using CLC Genomics Workbench 11.0 (QIAGEN, Aarhus, Denmark). For extraction of the consensus sequence, we selected an *Acropora* sp.1 sample (ID: Asp1S2003) with the highest short read coverage. A threshold of 10 was set for consensus sequence extraction, which is considered low coverage. Since consensus based on mapped reads cannot be generated in regions that meet or fall below 10, N was simply added to each base in the low-coverage region. Read conflicts were addressed using ambiguity codes representing all read bases represented at the reference position. The consensus sequence is shown in the supplemental material. The evolutionary divergence between the *WDR59* sequence of *A. digitifera* (adig_s0048.g28.t1) and *Acropora* sp. 1 (extracted consensus sequence) is estimated by MEGA 7. For estimates of evolutionary divergence between the two sequences, we selected the Kimura 2-parameter model (Kimura 1980) and a gamma distribution. There were a total of 2,895 positions in the final dataset.

**1.5.4 Phylogenetic three construction of the *WDR59* in *Acropora* species**

To confirm the orthologous relationship of *Acropora* sp. 1 and *A. digitifera WDR59*, we constructed a phylogenetic tree of *WDR59* using 14 *Acropora* species (*A. tenuis, A. intermedia, A. gemmifera, A. awi, A. florida, A. selago, A. hyacinthus, A. muricata, A. echinata, A. acuminata, A. nasuta, A. microphthalma, A. digitifera,* and *Acropora* sp.1). To avoid reducing the number of sites for phylogenetic analysis, *WDR59* sequence of *A. cytherea* (610 aa) and *A. yongei* (642 aa) were removed from the tree construction because these sequences were too short compared with *WDR59* of *A. digitifera* (964 aa). WDR24 sequence of *A. cytherea* (531 aa), *A. Florida* (675 aa), *A. yongei* (604 aa and 630 aa), and *A. tenuis* (606 aa) were removed from the tree construction because these sequences were too short compared with WDR59 of *A. digitifera* (824 aa). In total, 31 sequences from 14 *Acropora* species were used for phylogenetic construction. Gene ID used for the phylogenetic analysis were shown in table S7 and S8. We used a consensus sequence, explained above, for *WDR59* sequence of *Acropora* sp. 1 (1.5.3 Extraction of the consensus sequence of *WDR59* from *Acropora* sp. 1). We aligned *WDR59* sequences by MUSCLE with default parameters using MEGA X (Kumar, et al. 2018). We calculated the best-fitting nucleotide substitution model in MLModelTest by MEGA X (Kumar, et al. 2018). We selected the Jones-Taylor-Thornton (JTT) model and a gamma distribution with the lowest BIC score for further phylogenetic analysis by MEGA X(Kumar, et al. 2018) . We constructed a molecular phylogenetic tree of *WDR59* among 15 *Acropora* species using the Maximum Likelihood method. The complete deletion option was applied to eliminate positions containing gaps and missing data resulting in a final data set comprising 627 positions.

To determine whether three amino acid differences in *WDR59* between *A. digitifera* and *Acropora* sp. 1 are shared with other species or are specific to *Acropora* sp. 1, we aligned WDR59 amino acid sequences from 16 *Acropora* species by MUSCLE with default parameters using MEGA X(Kumar, et al. 2018) . A *WDR59* sequence of *A. millepora* was downloaded from the Kyoto Encyclopedia of Genes and Genomes (KEGG) for this alignment. The alignment result is shown in Figure S3. To determine whether the deletion in *WDR59* between *A. digitifera* and *Acropora* sp. 1 are shared with other species or are specific to *Acropora* sp. 1, we aligned WDR59 nucleotide sequence from 16 Acropora species by ClustalW with default parameters using MEGA X(Kumar, et al. 2018) . A *WDR59 sequence of A. millepora* was downloaded from the Kyoto Encyclopedia of Genes and Genomes (KEGG) for this alignment. The aligbment result is shown in Figure S4.

1.5.5 Gene expression levels of candidate genes

The transcriptome data of *A. digitifera* (SRR3316332, SRR3316333, SRR3316336, and SRR3316337) and *A. tenuis* (DRR288026-DRR288034) were downloaded from DDBJ. Downloaded raw reads were trimmed in the same manner as described above (1.2.1 Short reads alignment). Trimmed reads were imported to CLC Genomics Workbench 11.0 (QIAGEN, Aarhus, Denmark), and RNA-seq analysis was conducted with the following conditions; mapping type = map to gene regions only; mismatch cost =2; insertion cost =3; deletion cost =3; length fraction =0.9; similarity fraction =0.9; global alignment =No; auto-detect paired distance =yes; count paired reads as two =yes; use EM estimation =yes. For the transcriptome of *A. digitifera* and *A. tenuis*, we used *A. digitifera* genome assembly ver. 2.0 and *A. tenuis* genome assembly ver. 1.0 as reference genomes. To identify orthologs in *A. tenuis* of candidate genes identified in *A. digitifera*, we used the results of phylogenetic based OrthoFinder(Emms and Kelly 2019) using15 transcriptome of *Acropora* (explained in 1.5.2 Identification and alignment of WDR59 in other *Acropora* species). We manually extracted orthogroups containing 60 candidate genes of *A. digitifera* from the results of phylogenetic based OrthoFinder, and designated the *A. tenuis* genes included in those orthogroups as putative orthologs. Gene expression values (Transcripts per million: TPM) of 60 candidate genes were extracted from RNA-seq mapping results (Table S7 and S8).”

**2 Supplemental Figures**

**Figure S1.** In the HDRs, 60 genes harbor diverged SNVs. Among the 60 genes, 6 genes had putative orthologs in all four species (*Saccharomyces cerevisiae, Drosophila melanogaster, Danio rerio,* and *Homo sapiens*), 10 genes had putative orthologs in *Drosophila melanogaster, Danio rerio,* and *Homo sapiens*, 15 genes did not meet the aforementioned two criteria but had putative orthologs in at least one of the three model organisms (*Drosophila melanogaster, Danio rerio, and Homo sapiens*), and 29 genes had no orthologs in any of the four model organisms.

**Figure S2.** Phylogenetic relationship of the consensus sequence of the *WDR59* extracted from *Acropora* sp. 1. Phylogenetic relationships of *WDR59* and WDR24 in 14 *Acropora* species were analyzed based on 627 positions using Jones-Taylor-Thornton (JTT) model and a gamma distribution. Bootstrap support, shown next to each node for each clade, was obtained from 1000 replicates. Gene ID or accession number of genes used for the phylogenetic tree were shown in table S6 and S7.

**Figure S3.** Alignment of WDR59 amino acid sequences from 16 *Acropora* corals. Three amino acid sites that differ between *A. digitifera* and *Acropora* sp.1 are shown in blue.

**Figure S4.** Alignment of some *WDR59* sequences from *A. digitifera* and *Acropora* sp. 1 with the *A. digitifera* reference genome. Non-synonymous sites are shown in gray. The deletion is shown with a black line. Primer sequence locations are indicated by squares on the reference genome.

**Figure S5.** Alignment of *WDR59* sequences from 16 *Acropora* corals and the *Acropora* sp.1 *WDR59* sequence determined by PCR and sequencing.

**Figure S6.** A schematic diagram of the alignment of some *WDR59* sequences from 16 *Acropora* corals. Mutations shared with *Acropora* sp. 1 and *A. nasuta* are shown in blue. The mutation specific to *Acropora* sp. 1 is indicated by arrows.

**Figure S7.** Alignment of *A. digitifera* *WDR59* (Gene ID: adig_s0048_g28) as a query and template sequence (c8adlQ) using phyre2. Insertions relative to the template are shown in red. Deletions relative to the template are shown in orange. A black line surrounds the position of the *Acropora* sp. 1-specific mutation.

**Figure S8.** Gene expression (TPM) of *WDR59 in A. digitifera* during spawning. The x-axis indicates the transcriptome accession number and transcriptome information, and the y-axis indicates the gene expression level (TPM).

**
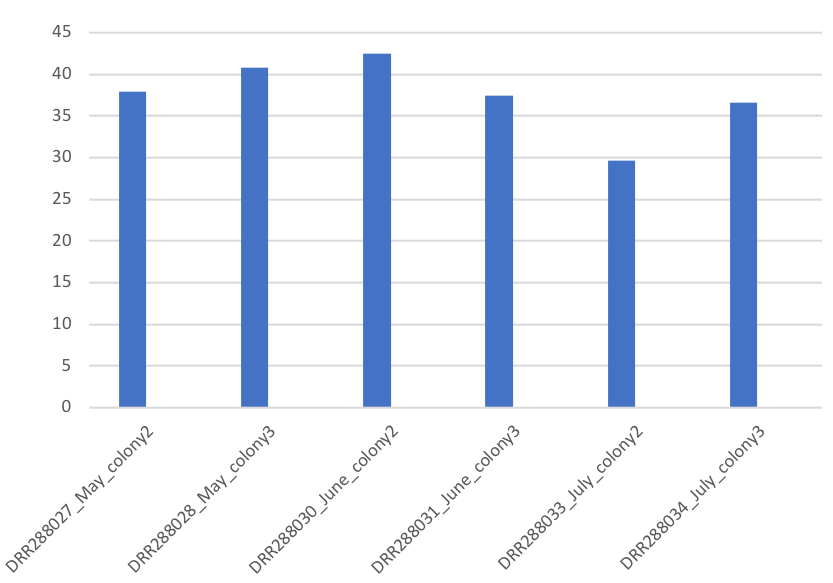
**

**Figure S9.** Gene expression (TPM) of *WDR59 in A. tenuis* during the spawning season. The x-axis indicates the transcriptome accession number and transcriptome information, and the y-axis indicates the gene expression level (TPM).

**3 Supplemental Tables**

Table S1. Sample for A. digitifera and Acropora sp. 1 nformation used in this study.

Table S2. Sample used in this study.

Table S3. The genomic location of HDRs in the *A. digitifera* genome assembly ver 2.0.

Table S4. The result of a Blastn search against the NCBI database using 29 genes as queries.

Table S5. Non-synonymous diverged SNVs in candidate gene

Table S6. Result of transcriptome cleaning

Table S7 Putative orthologs of WDR59 in Acropora

Table S8 Putative orthologs of WDR24 in *Acropora*

Table S9 Expression value of 60 candidate genes in *A. digitifetra*

TableS10 Expression values of 60 candidate genes in *A. tenuis.*

d SNVs in candidate genes.

Table S6

Table S5. Result of transcript clean up

Table S6 Orthologous genes of WDR59.

Table S7 Orthologous genes of WDR24.

Table S7 Eexpression values (TPM) of candidate genes using transcriptome of *A. digitifera* during spawning

Table S8 Eexpression values (TPM) of candidate genes using transcriptome of *A. tenuis* during the spawning season

**4 Supplemental Data**

4.1 Nucleotide sequences for identification of a deletion in WDR59

>AdigS1601

ACATCCGGGATATCCAGACACTAGGAATGTTGTCATGTGTGCTTGCGCATCACGCCCTTTCAGACGCTTTCAAACCTCCCAGAAACAGCTTTAACACTGAGGTGATTTCATCAAGCATGTCATTTCCTTTTGGCTCTCCATCGTCCTTTAACAACGATACTCCCTCACAGGTAATATTACAGC

>AdigS1602

TTTTCGTCCTGCACAGGATGGACTATTATTTGAACATCCGGGATATCCAGACACTAGGAATGTTGTCATGTGTGCTTGCGCATCACGCCCTTTCAGACGCTTTCAAACCTCCCAGAAACAGCTTTAACACTGAGGTGATTTCATCAAGCATGTCATTTCCTTTTGGCTCTCCATCGTCCTTTAACAACGATACTCCCTCACAGGTAATATTACAGC

>AdigS1603

TTTTCGTCCTGCACAGGATGGACTATTATTTGAACATCCGGGATATCCAGACACTAGGAATGTTGTCATGTGTGCTTGCGCATCACGCCCTTTCAGACGCTTTCAAACCTCCCAGAAACAGCTTTAACACTGAGGTGATTTCATCAAGCATGTCATTTCCTTTTGGCTCTCCATCGTCCTTTAACAACGATACTCCCTCACAGGTAATATTACAGC

>AdigS1604

TTTTCGTCCTGCACAGGATGGACTATTATTTGAACATCCGGGATATCCAGACACTAGGAATGTTGTCATGTGTGCTTGCGCATCACGCCCTTTCAGACGCTTTCAAACCTCCCAGAAACAGCTTTAACACTGAGGTGATTTCATCAAGCATGTCATTTCCTTTTGGCTCTCCATCGTCCTTTAACAACGATACTCCCTCACAGGTAATATTACAGC

>AdigS1606

TTTTCGTCCTGCACAGGATGGACTATTATTTGAACATCCGGGATATCCAGACACTAGGAATGTTGTCATGTGTGCTTGCGCATCACGCCCTTTCAGACGCTTTCAAACCTCCCAGAAACAGCTTTAACACTGAGGTGATTTCATCAAGCATGTCATTTCCTTTTGGCTCTCCATCGTCCTTTAACAACGATACTCCCTCACAGGTAATATTACAGC

>AdigS1607

TTTTCGTCCTGCACAGGATGGACTATTATTTGAACATCCGGGATATCCAGACACTAGGAATGTTGTCATGTGTGCTTGCGCATCACGCCCTTTCAGACGCTTTCAAACCTCCCAGAAACAGCTTTAACACTGAGGTGATTTCATCAAGCATGTCATTTCCTTTTGGCTCTCCATCGTCCTTTAACAACGATACTCCCTCACAGGTAATATTACAGC

>AdigS1610

TTTTCGTCCTGCACAGGATGGACTATTATTTGAACATCCGGGATATCCAGACACTAGGAATGTTGTCATGTGTGCTTGCGCATCACGCCCTTTCAGACGCTTTCAAACCTCCCAGAAACAGCTTTAACACTGAGGTGATTTCATCAAGCATGTCATTTCCTTTTGGCTCTCCATCGTCCTTTAACAACGATACTCCCTCACAGGTAATATTACAGC

>Asp1c

TTTTCGTCCTGCACAGGATGGACTATTATTTGAACATCCGGGATATCCAGACACTAGGAATGTTGTCATGTGTGCTTGCGCATCACGCCCTTTCAGACGCTTTCAAACCTTCCAGAAACAGCTTTAACACTGAGGAGATTTCATCAAGCACGTCCTTTAACAATGATACTCCCTCACAGGTAATATTACAGC

>Asp1B1901

TTTTCGTCCTGCACAGGATGGACTATTATTTGAACATCCGGGATATCCAGACACTAGGAATGTTGTCATGTGTGCTTGCGCATCACGCCCTTTCAGACGCTTTCAAACCTTCCAGAAACAGCTTTAACACTGAGGAGATTTCATCAAGCACGTCCTTTAACAATGATACTCCCTCACAGGTAATATTACAGC

>Asp1B1902

TTTTCGTCCTGCACAGGATGGACTATTATTTGAACATCCGGGATATCCAGACACTAGGAATGTTGTCATGTGTGCTTGCGCATCACGCCCTTTCAGACGCTTTCAAACCTTCCAGAAACAGCTTTAACACTGAGGAGATTTCATCAAGCACGTCCTTTAACAATGATACTCCCTCACAGGTAATATTACAGC

>Asp1B1903

TTTTCGTCCTGCACAGGATGGACTATTATTTGAACATCCGGGATATCCAGACACTAGGAATGTTGTCATGTGTGCTTGCGCATCACGCCCTTTCAGACGCTTTCAAACCTTCCAGAAACAGCTTTAACACTGAGGAGATTTCATCAAGCACGTCCTTTAACAATGATACTCCCTCACAGGTAATATTACAGC

>Asp1B1904

TTTTCGTCCTGCACAGGATGGACTATTATTTGAACATCCGGGATATCCAGACACTAGGAATGTTGTCATGTGTGCTTGCGCATCACGCCCTTTCAGACGCTTTCAAACCTTCCAGAAACAGCTTTAACACTGAGGAGATTTCATCAAGCACGTCCTTTAACAATGATACTCCCTCACAGGTAATATTACAGC

>Asp1B1905

TTTTCGTCCTGCACAGGATGGACTATTATTTGAACATCCGGGATATCCAGACACTAGGAATGTTGTCATGTGTGCTTGCGCATCACGCCCTTTCAGACGCTTTCAAACCTTCCAGAAACAGCTTTAACACTGAGGAGATTTCATCAAGCACGTCCTTTAACAATGATACTCCCTCACAGGTAATATTACAGC

>Asp1B1906

TTTTCGTCCTGCACAGGATGGACTATTATTTGAACATCCGGGATATCCAGACACTAGGAATGTTGTCATGTGTGCTTGCGCATCACGCCCTTTCAGACGCTTTCAAACCTTCCAGAAACAGCTTTAACACTGAGGAGATTTCATCAAGCACGTCCTTTAACAATGATACTCCCTCACAGGTAATATTACAGC

>Asp1B1907

TTTTCGTCCTGCACAGGATGGACTATTATTTGAACATCCGGGATATCCAGACACTAGGAATGTTGTCATGTGTGCTTGCGCATCACGCCCTTTCAGACGCTTTCAAACCTTCCAGAAACAGCTTTAACACTGAGGAGATTTCATCAAGCACGTCCTTTAACAATGATACTCCCTCACAGGTAATATTACAGC

>Asp1S2001

TTTTCGTCCTGCACAGGATGGACTATTATTTGAACATCCGGGATATCCAGACACTAGGAATGTTGTCATGTGTGCTTGCGCATCACGCCCTTTCAGACGCTTTCAAACCTTCCAGAAACAGCTTTAACACTGAGGAGATTTCATCAAGCACGTCCTTTAACAATGATACTCCCTCACAGGTAATATTACAGC

>Asp1S2002

TTTTCGTCCTGCACAGGATGGACTATTATTTGAACATCCGGGATATCCAGACACTAGGAATGTTGTCATGTGTGCTTGCGCATCACGCCCTTTCAGACGCTTTCAAACCTTCCAGAAACAGCTTTAACACTGAGGAGATTTCATCAAGCACGTCCTTTAACAATGATACTCCCTCACAGGTAATATTACAGC

>Asp1S2003

TTTTCGTCCTGCACAGGATGGACTATTATTTGAACATCCGGGATATCCAGACACTAGGAATGTTGTCATGTGTGCTTGCGCATCACGCCCTTTCAGACGCTTTCAAACCTTCCAGAAACAGCTTTAACACTGAGGAGATTTCATCAAGCACGTCCTTTAACAATGATACTCCCTCACAGGTAATATTACAGC

>Asp1S2005

TTTTCGTCCTGCACAGGATGGACTATTATTTGAACATCCGGGATATCCAGACACTAGGAATGTTGTCATGTGTGCTTGCGCATCACGCCCTTTCAGACGCTTTCAAACCTTCCAGAAACAGCTTTAACACTGAGGAGATTTCATCAAGCACGTCCTTTAACAATGATACTCCCTCACAGGTAATATTACAGM

>Asp1S2007

TTTTCGTCCTGCACAGGATGGACTATTATTTGAACATCCGGGATATCCAGACACTAGGAATGTTGTCATGTGTGCTTGCGCATCACGCCCTTTCAGACGCTTTCAAACCTTCCAGAAACAGCTTTAACACTGAGGAGATTTCATCAAGCACGTCCTTTAACAATGATACTCCCTCACAGGTAATATTACAGC

>Asp1S2008

TTTTCGTCCTGCACAGGATGGACTATTATTTGAACATCCGGGATATCCAGACACTAGGAATGTTGTCATGTGTGCTTGCGCATCACGCCCTTTCAGACGCTTTCAAACCTTCCAGAAACAGCTTTAACACTGAGGAGATTTCATCAAGCACGTCCTTTAACAATGATACTCCCTCACAGGTAATATTACAGM

4.2 **consensus sequence of *WDR59* from *Acropora* sp. 1**

>Acroporasp1_WDR59_cds_consensus_Asp1S2003

atgttgactgacgatatccatcctgaaaccaagatggcgtcttaccagaacgcagttgca

gtgcacgactggcctgcaacagtcatggctgtggactgtastggtcattttactgtcctt

ggatcgagaaaaggattggcattcattgacttgaactctcccastgttatcacaaaaaag

gtgccaaggaacagcaaatgggaatgtaatgccctggaatggaatcctcacstttcggat

gctcatatctttgctaatgcttcaaatcaaaaaacagaaatctggtcttggagtaatggc

aatggattacagctgcaaattcttagaggtcatacaagagcaattagtgatctaaactgg

tcttggtttgaccctcagctactgtccagctgctcaatggatcagtttatatacatttgg

gacttgagggagggtaaaaagccagctagctcattgcaagctattgttggtgcctcacaa

gtgaaatggaacagagtaaaccggcatgtccttgcaacaagccatgatggagatgttcgg

atctgggatcttaggaaaggtaacacaccygtgatctacctaacagcccacttgtctaag

attcatggtcttgattggtcacgctccagtggcacaactctggcaacttgcagtagtgat

actaccgtcaagttgtggaatactgaacaacctcagcaaccagagaakaagttgaatgca

aagtgccctgtctggagagcaagatttacaccatttggagagggacttgttactgtaact

ctaccacaattacagcagggagagaactacttgtctctgtggaacattccagatgtcaac

tctccagtggccgcacctgtcaacacttttgtcggccacagtgatgtggtgttggacttt

cattggagatcccaaactcttgatraagatgagcagtttcagcttatcacttgggcaaag

gactgttgtcttcgtttatgggttcttgagccaaggatgattatggcatgcagtggtgat

acatccaccaatttcccttctcgttcaaccaatgaaacagcccactccatgtctgaggag

ctrataaagatggcaannnnngaacacctgatcttgcagcacagccacagacattacagc

aggaattcgctctcattaatgtcaacattccaaatgtcactgttgagcagttggatgctg

ggcacaggagctgtactgtgtgtgctacaagtggacccgatactgtttccttggtgataa

actttccctccctgtaccctaaccaagccatcccatcatttgagtttacagcggacacct

cgattgataccaatactaagacaaagctaatgaagaccctatgtgaaacagctcagactc

atgtgaaattaaaccaaacttgtcttgaaccctgtctgaggcagctggtcagccatcttg

aggaactaacgcttcaagaacgattacccatggatcagtttgccgctactgcgcctcaac

tgcatgaccgactcgatccaatctgttacgttagctttcttgacgcagctattcctttcc

cacgcacatctggcgcgaaattctgtgcttgtggattgctggtgtgttttaatttgccgg

gaagatacggtggccgggtggggtctggtggagaaccgactccaagatctctatcagctt

tctcggcatacagttctcgccctagtagtggcccggctcttccccgacttatcaacagac

attttcaaaagcctctccaaagacatactttcggttcgtttcgccagctgacaaggacct

tcacgtacagagctttaagcaggacagaagacccacaggatagacaaaagtacagtgggt

taccctctaggaacagtgatgttggcctggttgtgattcgtgacgtcagtgctatgatgc

ccatccatcagtcgctgggtcaggcatacacgttggaagggaacaatatcactaagattt

gcaagaaaaacctgtctgctgccatgacaacgggacgaaaagacctggtgcagacatggt

ctcttttgagcttagtgctcgatgagaagctggctccctcagactccatcgacgaggcgc

cctgggcgctgcatccatttggcaagaaattagtcagttccctgatggactattatttga

acatccgggatatccagacactaggaatgttgtcatgtgtgcttgcgcatcacgcccttt

cagacgctttcaaaccttccagaaacagctttaacactgaggnnnnnnnnnnnnnnnnnn

nnnnnnnnnnnnnnnnnnnnnnnnnnnnnnnnnnnnnnnnnnnnnnnnnnnnnnnnnnnn

nnnnnnnnnnnnnnnnnnnnnnnnnnnnnnnnnnnnnnnnnnnnnnnnnnnnnnnnnnnn

nnnnnnnnnnnnnnnnnnnnnnnnnnnnnnnnnnnnnnccaacagtttttattcgaagta

aggatccaatggaagaacaaaaaaagcagtttaaaagtgactgcagatttgtcgaccctt

cgcaggtcaaactgcatgaccattacataaaacagtacgctgatgttctgtaccgatgga

gtttgctgggcaaaagagctgaagttaccaaattcttaagtgaaccgcaattgcctcaca

gtggcgcagaattcagtacgcgttgctacaattgttcccgaaaccttcgtggtgctcagt

gtggttcttgtaaatcgtttggactacagtgtgtgatatgtcatgtggctgtaagaggtg

ccagtaatttctgtgtcgcctgcggtcacggcggtcacgcgtatcatctattgacgtggt

ttaaatctatggatgtctgcccaacaggctgtggatgtaggtgtcgggaagtcggtacat

tcattgtggattga

**References**

Altschul SF, Gish W, Miller W, Myers EW, Lipman DJ 1990. Basic local alignment search tool. J Mol Biol. 215: 403-410.

Chen S, Zhou Y, Chen Y, Gu J 2018. fastp: an ultra-fast all-in-one FASTQ preprocessor. Bioinformatics 34: i884-i890.

Chiu Y-L, Shikina S, Yoshioka Y, Shinzato C, Chang C-F 2020. De novo transcriptome assembly from the gonads of a scleractinian coral, Euphyllia ancora: molecular mechanisms underlying scleractinian gametogenesis. BMC Genomics 21: 1-20.

Danecek P, et al. 2011. The variant call format and VCFtools. Bioinformatics 27: 2156-2158.

Danecek P, et al. 2021. Twelve years of SAMtools and BCFtools. Gigascience 10: giab008.

DePristo MA, et al. 2011. A framework for variation discovery and genotyping using next-generation DNA sequencing data. Nature genetics 43: 491-498.

Emms DM, Kelly S 2019. OrthoFinder: phylogenetic orthology inference for comparative genomics. Genome biology 20: 1-14.

Hudson RR, Slatkin M, Maddison WP 1992. Estimation of levels of gene flow from DNA sequence data. Genetics 132: 583-589.

Kimura M 1980. A simple method for estimating evolutionary rates of base substitutions through comparative studies of nucleotide sequences. Journal of molecular evolution 16: 111-120.

Kumar S, Stecher G, Li M, Knyaz C, Tamura K 2018. MEGA X: molecular evolutionary genetics analysis across computing platforms. Molecular Biology and Evolution 35: 1547-1549.

Kumar S, Stecher G, Tamura K 2016. MEGA7: Molecular Evolutionary Genetics Analysis version 7.0 for bigger datasets. Molecular Biology and Evolution 33: 1870-1874.

Langmead B, Salzberg SL 2012. Fast gapped-read alignment with Bowtie 2. Nature methods 9: 357-359.

McKenna A, et al. 2010. The Genome Analysis Toolkit: a MapReduce framework for analyzing next-generation DNA sequencing data. Genome research 20: 1297-1303.

Pfeifer B, Wittelsbürger U, Ramos-Onsins SE, Lercher MJ 2014. PopGenome: an efficient Swiss army knife for population genomic analyses in R. Molecular Biology and Evolution 31: 1929-1936.

Poplin R, et al. 2017. Scaling accurate genetic variant discovery to tens of thousands of samples. bioRxiv: 201178.

Shinzato C, et al. 2021. Eighteen coral genomes reveal the evolutionary origin of Acropora strategies to accommodate environmental changes. Molecular Biology and Evolution 38: 16-30.
